# Thermo-Electro-Mechanical Simulation of Semiconductor Metal Oxide Gas Sensors

**DOI:** 10.3390/ma12152410

**Published:** 2019-07-28

**Authors:** Lado Filipovic, Siegfried Selberherr

**Affiliations:** Institute for Microelectronics, TU Wien, Gußhausstraße 27-29/E360, 1040 Vienna, Austria

**Keywords:** gas sensors, semiconductor metal oxide, modeling and simulation, electro-thermo-mechanical modeling, finite element method, CMOS fabrication, MEMS membrane, microheater, hotplate, Joule effect

## Abstract

There is a growing demand in the semiconductor industry to integrate many functionalities on a single portable device. The integration of sensor fabrication with the mature CMOS technology has made this level of integration a reality. However, sensors still require calibration and optimization before full integration. For this, modeling and simulation is essential, since attempting new, innovative designs in a laboratory requires a long time and expensive tests. In this manuscript we address aspects for the modeling and simulation of semiconductor metal oxide gas sensors, devices which have the highest potential for integration because of their CMOS-friendly fabrication capability and low operating power. We analyze recent advancements using FEM models to simulate the thermo-electro-mechanical behavior of the sensors. These simulations are essentials to calibrate the design choices and ensure low operating power and improve reliability. The primary consumer of power is a microheater which is essential to heat the sensing film to appropriately high temperatures in order to initiate the sensing mechanism. Electro-thermal models to simulate its operation are presented here, using FEM and the Cauer network model. We show that the simpler Cauer model, which uses an electrical circuit to model the thermo-electrical behavior, can efficiently reproduce experimental observations.

## 1. Introduction

The way in which we perceive the environment is heavily influenced by the presence of various gases in our vicinity. The human nose works to detect a large variety of different smells, but it fails outright in the detection of many harmful gases or in the detection of a specific concentration of a target gas. The ability to measure the exact quantity of useful and harmful gases in our vicinity has been a topic of high interest for several decades. In fact, prior to the rise of electronics and microelectronics, the capabilities of animals were used for sensing certain gases. An obvious example is the use of a canary as a detector of harmful gases in mines; the canary–a songful bird–stops singing when exposed to methane, carbon dioxide, or carbon monoxide, signaling to miners the presence of harmful air. Many industries and applications depend on gas sensors such as environmental monitoring [1,2,3], health and safety [4], automotive [5,6,7], and chemical warfare detection [8,9,10]. The main goal of gas sensor research today is to allow for its miniaturization and reduced power consumption for integration with portable electronics.

The aggressive transistor scaling, the primary goal of the semiconductor industry for many years, has been the principal enabler of today’s hand-held and wearable devices. This trend along Moore’s Law [11] is powered by advancements in complementary metal oxide semiconductor (CMOS) fabrication. More recently, there has been a drive to integrate more devices on a single chip; this means the integration of more than just transistors, but also multiple applications, such as sensors or radio frequency (RF) circuits, labeled as the More-than-Moore approach [12,13]. The first attempts at integration of multiple functionalities were on the package level and this in-package integration dealt with connecting multiple dies with bond wires and packaging them together. This method negatively impacts performance and power dissipation due to the need for long wires, which results in a higher circuit resistance and thereby in increased resistance/capacitance (RC) delays. A better alternative for connecting micro-electro-mechanical systems (MEMS) applications with CMOS technology is using three-dimensional (3D) integration. With this technique, the dies with different functionalities are stacked on top of each other, thereby eliminating the need for long bonding wires and allowing for a straightforward single packaging step. Ultimately, the ideal integration of CMOS and MEMS features is to have all the necessary structures, materials, and processing technologies available within a CMOS fabrication facility and on a single wafer. CMOS technology has the advantage of being a mature and cost efficient technology, which is readily available and allows for fabless companies to concentrate on design only. Furthermore, the integration of electronics together with MEMS features required in a gas sensor becomes intuitive, since all components are fabricated within a CMOS foundry.

In this review we discuss recent achievements in the modeling of integrated SnO${}_{2}$ gas sensors within an advanced CMOS technology. The temperature used for the fabrication of the MEMS components should not exceed 450 ${}^{\circ }$C in order to not damage the front end of line (FEOL) components, such as the transistors. After the introduction, the manuscript is divided into four main sections: The first one is meant to convey to the reader the significance of the semiconductor metal oxide (SMO) gas sensor and what its primary advantages are over alternate gas sensor solutions, which become very clear. Futhermore, the SMO gas sensor and its composition is introduced, while the second section looks at the means to model the SMO sensor structure, which includes a microheater and membrane element, concentrating on fabrication and electro-thermo-mechanical simulations using the finite element method (FEM). Here we also look at the material stacks used to generate the suspended membrane and the essential aspects for its modeling and simulation. The third section deals with how the electro-thermal response is modeled and its applied bias-temperature-power relationship, while avoiding complex meshes required in FEM simulations. A deep analysis through modeling and simulation has enabled the development of optimized devices with significant improvements in design and power dissipation for advanced sensors and microheaters. In the subsequent section, recent achievements in understanding and modeling the sensing behavior of SMO films, including its conduction and surface adsorption mechanisms, are described. Finally, the manuscript is summarized in the conclusion. Before discussing the described sections, the following subsections discuss achievements in the fabrication of SMO gas sensors, which are primarily geared towards the deposition of the microheater and the SMO sensing layer.

### 1.1. Micro-Hotplate Fabrication

Several processing steps are required in order to fabricate the SMO sensor, but the two which are the most challenging are the formation of the membrane, or membrane release, and the deposition of the sensing film. The quality of the membrane with microheater and SMO film define the sensor performance and stability, which is why their careful fabrication is of utmost importance. The key concern with membrane release is that the process should be compatible with CMOS technology to ensure cost-efficient fabrication. This involves the proper choice of membrane, microheater, and adhesion materials [14]. For electrical isolation and CMOS compatibility, silicon dioxide (SiO${}_{2}$) and silicon nitride (Si${}_{3}$N${}_{4}$) are the most commonly used membrane materials. The microheater layer is either deposited on top of the membrane in a coplanar design or is sandwiched between the nitride and oxide films in a buried approach. In either case the proper adhesion of the microheater film to these layers is essential. Recently, the electrical and thermal behavior of the coplanar and buried approaches were compared and a suitable fabrication strategy for both was proposed [15]. The buried hotplate is primarily used when a uniform temperature needs to be provided to the sensing area, meaning that a single operating temperature at a time is desired. Alternatively, the coplanar microheater integration can provide a very linear temperature gradient across the active region, which has a potential application in gas sensor arrays [15]. Since the temperature influences sensing, having multiple temperatures available for simultaneous read-out helps improve the selectivity of the gas sensor. An alternative approach to the coplanar heater is a microheater array [16]. With a microheater array, a single layer is used to provide several predictable temperatures to desired regions. This is achieved by thinning certain sections of the metal line to ensure a desired temperature profile therein [17].

Finding an appropriate choice for the microheater material is addresssed in some detail in Section 4.1, where we describe several materials currently under investigation. However, platinum is currently the most frequently used material for microheaters in gas sensor applications, which is mainly due to it being chemically inert up to high temperatures. The micro-hotplate provides many advantages, including miniaturized size, high sensitivity, low power consumption, fast response, and adjustable temperature to improve selectivity for the gas sensors. The deposition of a platinum film is usually preceded by the deposition of an adhesion layer which ensures that the platinum film sticks to the isolation silicon dioxide or silicon nitride films which make up the membrane, reducing the chance of delamination [18]. Many adhesion layers are inadequate because, although they adhere to both the platinum and silicon nitride layers, they tend to diffuse into the platinum during high temperature operation, thereby adversely changing the electrical behavior of the platinum heater. These include chromium (Cr), titanium (Ti), tungsten (W), nickel (Ni), iron (Fe), and tantalum (Ta) [19]. The temperature at which diffusion takes place is different for each adhesion metal. The authors in [18] found that pre-oxidation of the adhesion layer reduces diffusion during later high-temperature processing or operation. They also note that Ti diffuses into the platinum much more frequently than Ta or zirconium (Zr). Titanium and tantalum are frequently used as dielectrics and adhesion layers for films in CMOS fabrication [20], which is why it would be beneficial if one of those could be applied to the microheater deposition as well. In [14], the authors studied the behavior of sputtered Ta and Ti as adhesion layers for platinum. They found that using Ti resulted in much smaller grains in the platinum, when compared to using Ta or without any adhesion layer. In addition, using Ta or no adhesion layer results in less variation in Pt surface mobility and no difference could be seen regardless if the layer was annealed in N${}_{2}$ or O${}_{2}$ ambient, which was not the case when Ti adhesion was used. The observed continuous structural degradation of Pt films above 500 ${}^{\circ }$C suggests an upper limit for its use as a microheater in gas sensor applications. Interestingly, the use of an adhesion layer could be avoided altogether by roughening the Si${}_{3}$N${}_{4}$ surface prior to Pt deposition [14].

### 1.2. Sensing Film Deposition

Many techniques have been successfully applied over the last decades to deposit thick and thin semiconductor metal oxide films for gas sensing devices. The primary goal of the type of film used is to ensure the highest sensitivity and selectivity. The sensitivity is introduced by maximizing the film’s surface-to-volume ratio. This is achieved even in thick porous films by their granular nature. The grains inside the film expose their entire surface to the ambient gas, making sure that most of the film’s volume is also used for sensing and not only the surface which in direct contact with the ambient [21,22,23]. Other means of introducing a higher surface to volume ratio involves the fabrication of nanosized sensing elements, including nanofilms [24], nanowires [25], nanorods [26], nanoparticles [27], nanocrystals [28], nanofibers [29], nanospheres [30], nanotubes [31], nanoplates [32], and nanopowders [33].

**Figure 1:**
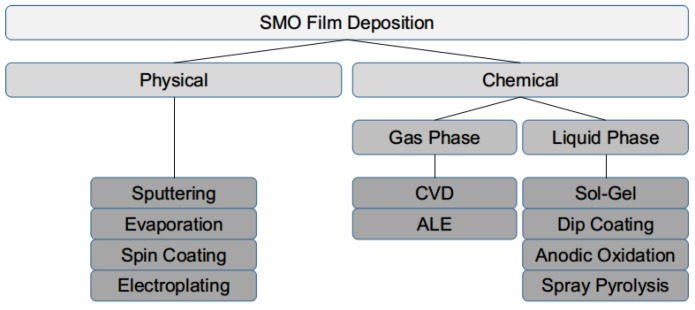
Summary of techniques used for SMO film deposition, where CVD is chemical vapor deposition and ALE is atomic layer epitaxy.

Some of the techniques recently used to deposit SMO films are shown in Figure 1. Of primary importance is that the deposition can proceed in a way conducive for integration with CMOS foundry technology. Recent studies also investigated ink-jet printed SMO films for gas sensing with some success [34]. However, ink-jet printing is not ideal when integration with CMOS technology and mass production are desired. Sputtering [35,36], chemical vapor deposition (CVD) [37,38], and spray pyrolysis [39,40] have shown to be integrable with the CMOS process. Spray pyrolysis has garnered significant attention recently due to its cost-efficiency, which proceeds by heating the wafer to elevated temperatures (400 ${}^{\circ }$C) and spraying a liquid solution over it. The particles which reach the surface as a vapor then proceed to deposit a high quality film. The deposited thin film is very uniform around corners and edges, allowing for innovative geometries to be coated while requiring no expensive equipment or complex setup. Recently, a method to take advantage of the heating capability of the integrated microheater to deposit an SMO sensing film was described [37]. The method was used to deposit a layer of tungsten oxide (WO${}_{3}$) with and without added gold (Au) and platinum (Pt) functionalization. This method has the potential to fabricate microheater arrays with tunable properties without the use of additional masks or additional thermal processing steps. Furthermore, the localized heating means that a complete photolithography treatment, meaning the removal of the film where it is not desired, and the associated costs can be avoided.

## 2. SMO Gas Sensor

### 2.1. Gas Sensing Mechanisms

Recently, the push towards the Internet of Things (IoT) and Internet of Everything (IoE) has resulted in substantial research efforts being alloted for the development of portable gas sensors [41,42,43]. Portability can be achieved through integration with a mobile device, such as a smartphone, wrist- and smart-watches, tablets, or wearables. There are many different gas sensing principles under investigation today and some have already found success in industry and research. These include semiconducting materials, optical devices, thermal conductivity sensors, infrared (IR) detectors, quarz microbalance sensors, catalytic detectors, dielectric detectors, electro-chemical sensors, and electrolyte based sensors [44,45,46,47]. While all types have their advantages and disadvantages, the semiconducting metal oxide gas sensor appears to be the most appropriate choice for miniaturization, low power conduction, and thereby portable applications. Why this is the case is further discussed in this section and is summarized in Table 1, where the existing gas sensing technologies are evaluated based on several parameters including sensitivity, accuracy, selectivity, response time, stability, durability, power, cost, and footprint. The primary classification for gas sensor devices includes those whose sensing mechanism is based on a change in the electric properties of a film and those whose sensing mechanism is based on a variation of another material property, when a target gas is found in the ambient [48]. Table 1 stems from a collection of recent reviews from Korotcenkov [45], the updated discussion by Dey [46], as well as the updates and additions of photoionization and piezoelectric devices from Filipovic and Lahlalia [47].

From Table 1 we note that the SMO sensor has the highest rating for power, cost, and footprint. The key enabler of these ratings is the recently developed fabrication integrated in CMOS technology of these devices. These three components are essential for integration and portability. It should also be noted that infrared adsorption sensors, while exhibiting excellent operational properties, including sensitivity, accuracy, and selectivity, have concerns, when it comes to integration due to their high costs, footprint, and power dissipation; however, there is a concentrated effort to design micro-sized CMOS compatible IR adsorption sensors, which could be revolutionary. One of the problems with SMO sensors is that direct chemical interactions are required in order for sensing to take place, which is not the case for IR detection and would be a significant benefit, should these sensors ever reach the cost and power levels necessary for portable applications.

From Table 1 it is evident that SMO gas sensors have the most advantages, especially concerning portability, while selectivity is something which still needs to be improved. The repeatability and the reproducibility of SMO sensors is another advantage they exhibit, which is partly enabled due to their CMOS-friendly fabrication [49]. It is of critical importance that the same structure with a predictable geometry and predictable operating conditions is fabricated every single time when developing commercial devices. Without this, the mass production of these gas sensors would be hindered. A high level of certainty must exist in a commercial mass manufacturing process whenever it is not feasible to precisely test every individual device prior to its delivery to customers. Various semiconductor materials have been tested as gas sensors over the years, including most notably graphene, transition metal dichalcogenides, and other two-dimensional materials, but these are still at a research stage, since they do not have enough reproducibility to allow for mass industrial production. Therefore, SMOs are still quite dominant, when it comes to their availability and application in the semiconductor sensor market.

### 2.2. SMO Sensor

The gas sensing mechanism of SMO films is based on the film’s changing electrical behavior in the presence of a target gas. A film, such as tin dioxide (SnO${}_{2}$) will have its resistance change when exposed to a target gas and when heated to several hundred degrees Celsius. The high temperature gives the gas molecules enough energy to initiate a surface reaction which results in the exchange of charge carriers and ultimatly a change in the film’s conductive behavior. As is evident from Table 1, one of the main flaws of the SMO sensor is its selectivity, which can be understood by observing that a simple change in resistance does very little to identify precisely which gas molecule has adsorbed on the surface. As a workaround to this problem, the selectivity of the SMO sensor is introduced through a sensor array, similar to that of the piezoelectric sensor. The array provides simultaneous detection at multiple test conditions; this could mean multiple temperatures or differently doped sections of an SnO${}_{2}$ film. The sensors’ data then requires further post-processing using one of many potential methods, including neural networks or machine learning. Several challenges still remain to ensure that the SMO is fully integrated in portable electronics, including the aforementioned selectivity and improved mechanical reliability.

The main improvements still desired from SMO sensors include:

- The main concerns with the mechanical stability of SMO sensors is due to the need of high temperature operation of the metal oxide film. Regularly heating a device to temperatures between 250 ${}^{\circ }$C and 500 ${}^{\circ }$C from room temperature and then cooling them back down results in added mechanical stresses and poor stability in all involved layers. Allowing for a reduction in the operating temperature to levels below 100 ${}^{\circ }$C would lead to an improved stability, reliability, and power consumption.
- In order to operate at high temperatures, a microheater must be integrated underneath the sensing layer. Due to this requirement, thermal isolation must be provided from the surrounding devices, complicating the fabrication process and demanding a MEMS suspended membrane. Furthermore, the temperature provided has a large impact on the sensing response, while knowing the exact microheater behavior is not always possible, especially since the properties of the microheater materials change with time under operation. These changes can be brought up by the induced thermal stresses, thermo-migration, or electro-migration [47].
- The SMO’s selectivity is another concern. This is currently being addressed by introducing a sensor array, where multiple sensors are individually engineered to increase their selectivity towards a particular gas [50,51,52,53,54,55]. By combining many sensors, each with a prevalent response towards a particular gas, the collected data set can be post-processed to better pin-point which gas or gases are adsorbed at the surface [56]. The requirement of added post-processing makes efficient CMOS integration even more essential, since integration with CMOS electronics would allow the sensor to operate at increased speeds while reducing the power and signal losses readily associated with long interconnect lines.
- There are several research groups looking into the processes taking place during the SMO sensors’ operation; however, a full understanding is as of yet not available. Until recently, it was thought that sensing was only due to a redox surface reaction with adsorbed oxygen. However, it was not long ago shown that even when oxygen is not present, a thin accumulation layer can form at the SMO film’s surface. This layer is formed due to the direct adsorption of gas molecules by the surface oxygen vacancies and results in a change in the film’s resistivity. Many studies also show that adding a dopant to an SMO film can improve the sensitivity or selectivity towards desired gases. Modeling all the simultaneously-occurring phenomena, including dopant influences, is not currently available. Such a predictable model would be very beneficial towards developing a technology computer aided design (TCAD) environment for the design and optimization of SMO sensors.
- Due to the nature of the sensing mechanism, gas molecules can remain adsorbed to the surface even after a sensing cycle has already concluded. In industrial applications, annealing to higher temperatures in clean air or vacuum (above 500 ${}^{\circ }$C) promotes the removal of adsorbed species and surface contaminants; however, this is not feasible in portable electronics [57]. Ideally, the simple cooling to room temperature should result in the desorption of all species on the surface, but this is not the case and a removal procedure must be incorporated in the portable device. Removing the previous species is essential in order to ensure that all initially available surface adsorption sites are once again ready for the next measurement cycle.

**Figure 2:**
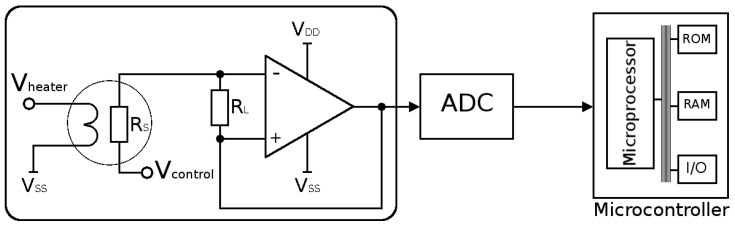
Schematic of an SMO sensor with interface blocks. The sensor includes a heating element and sensing film, a voltage follower, and an analog-to-digital converter (ADC). To process the sensor data, it is connected to a microcontroller, which contains a read-only-memory (ROM), random access memory(RAM), and input/output (I/O) interfaces.

The SMO sensor was found to be suitable in many applications, including medical, automotive, sensor networks, food quality monitoring, and wearable devices, making it a primary candidate for its use as a universal sensor [47,58,59]. A typical integrated SMO sensor circuit is given in Figure 2. The advantages, but also the complexity of its fabrication can imediately be seen. The final device is powered by a microheater (V${}_{\mathrm{heater}}$ in Figure 2), which heats a sensing layer (R${}_{S}$ in Figure 2), and additional analog, digital, and RF circuit components, all integrated on a single chip [60,61]. The encircled section corresponds to the sensor itself, which contains the heater element and the SMO layer which is the resistive sensing element. Several aspects can be identified from this figure:

- As can be expected, the surface of the sensitive SMO film must be exposed to the ambient in order to interact with the target gas molecules. Therefore, its deposition must be performed at the end of the CMOS front end of line sequence. This means that the deposition must proceed at low temperatures, not exceeding the typical back end of line (BEOL) fabrication temperature of about 400 ${}^{\circ }$C.
- A microheater element is the essential component used to heat the SMO layer locally to quite high temperatures to ensure enough energy is reached to initiate gas sensing. Here we concentrate on several modeling and simulation aspects for the membrane which is required to house the microheater/sensor element as well as the microheater itself.

### 2.3. Choice of Sensing Film

The ongoing miniaturization of electronics has shown to be essential for integration and advancing our technological capabilities. Transistor scaling has steadily followed Moore’s law up to the physical limitations; the gas sensor and environmental sensor fields have, nevertheless, lagged behind the progress achieved by CMOS devices, primarily due to their bulky nature and complex MEMS fabrication requirements. Discoveries in the application of semiconductor materials for gas sensing have given hope for the miniaturization, portability, and integration with the electronics of sensing systems. The first demonstration of the capability of a semiconductor to sense certain gas molecules, especially when heated, was performed by Brattain and Bardeen in the 1950s [62]. They noticed, for the first time, that the conductivity of several semiconductor films changed, when the chemical composition of the ambient was changed. The first gas sensing device based on this discovery was developed in the 1960s using zinc oxide (ZnO) as the sensing layer heated to 485 ${}^{\circ }$C [63]. To add to this dicsovery, by 1967 Shaver showed that doping the films slightly using noble metals, such as platinum, rhodium, iridium, gold, or paladium, can improve the sensor performance [64].

In the last decades, research into SMO materials has intensified and the search for the proper combination of dopants to improve the overall sensor sensitivity as well as its selectivity towards desired gases has been studied extensively. Some of the SMOs which have been researched for their gas sensing properties include nickel oxide (NiO), indium oxide (In${}_{2}$O${}_{3}$), indium tin oxide (ITO), cadmium oxide (CdO), zinc tin oxide (ZnSnO${}_{4}$), lead oxide (PbO), yttria-stabilized-zirconia (YSZ) and many more. Among the most promising are tin dioxide (SnO${}_{2}$), zinc oxide (ZnO), and tungsten trioxide (WO${}_{3}$). Tin dioxide has also been recently commercialized by several manufacturers [65,66,67,68,69,70]. SnO${}_{2}$ fulfills almost all of the key requirements for a good gas sensing film, which includes a sensitivity to many different gases, CMOS technology friendly deposition, and low fabrication costs [44]. The first SMO gas sensor was used for safety monitoring and was patented by Taguchi in the early 1970s. The device used a porous SnO${}_{2}$ film with a slight palladium doping [71,72]. In the five decades since the initial patent, many researchers have dedicated their efforts to finding the perfect metal oxide film. From recent studies, it appears that SnO${}_{2}$ is the clear favorite and is likely the best option due to it being able to detect almost all gases of relevance [44,73].

The sensing response of a SMO film is defined by the change in resistance of the film, when exposed to a target gas ($R_{g}$), compared to its baseline resistance in air ($R_{a}$):

(1) $$Sensitivity=\frac{R_{a}-R_{g}}{R_{g}}\cdot 100\%$$

**Figure 3:**
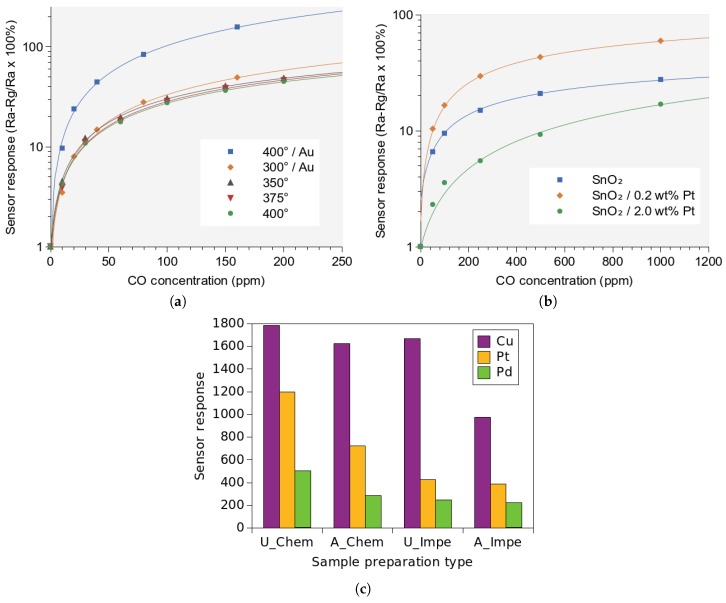
Sensing response of an SnO2 film after exposure to carbon monoxide from several publications. (**a**) Köck et al. [74,75] show the influence of temperature and gold doping on the operation of a 50 nm thin film. (**b**) Mädler et al. [77] show the role that platinum (Pt) doping plays on CO detection. (**c**) Tangirala et al. [78] study the influence of dopants and doping methods on the sensitivity of SnO${}_{2}$ films towards the detection of CO.

In Figure 3a the responses of an SnO${}_{2}$ film under exposure to CO in the environment is shown, assembled and summarized from several recent works from the group of Köck et al. [74,75]. The 50 nm thick film was deposited using a post-CMOS spray pyrolysis technique. The results are shown for several temperatures as well as with and without the influence of gold (Au) doping through an added 2 nm evaporated film. It is clear that the added gold particles help improve the response time significantly at 400 ${}^{\circ }$C while not having a large influence at 300 ${}^{\circ }$C. The best fit lines show that there is a logarithmic dependence on the reacting gas concentration. This is commonly observed when the reacting gas concentration is nearing saturation with respect to the number of available adsorption locations on the SMO surface. This also limits the capability of the sensor to detect small changes in the target gas concentration [76].

The results shown in Figure 3b are from measurements made on a Pt-doped SnO${}_{2}$ film with a thickness of 50 nm. The film was deposited using spray pyrolysis and the microheater temperature was set to 300 ${}^{\circ }$C during operation. [77]. An interesting pattern can be seen therein. When the platinum dopant was added to SnO${}_{2}$ at a concentration of 0.2 wt%, the sensor signal increased by about 10 times. However, when the platinum dopant concentration was increased by 10 times (from 0.2 wt% to 2.0 wt%), the sensor signal decreased. In fact, the signal decreased to levels below even the non-doped SnO${}_{2}$ film. The initial increase in the response in the presence of Pt particles takes place due to the dissociation of oxygen on platinum. The activated oxygen species are able to reach the SnO${}_{2}$ film, where they react with CO [77]. The reduced sensing response in the presence of 2 wt% Pt doped SnO${}_{2}$ can likely be attributed to a localized consumption of CO molecules without any donation or acceptance of electrons. This means that even in the presence of CO molecules, no changes in the SnO${}_{2}$ film’s resistivity can take place. Another reason may be because the added platinum particles shift the optimal operating temperature for the sensing film. While 300 ${}^{\circ }$C was appropriate for pure SnO${}_{2}$ films, the added platinum may have shifted the peak operating conditions to about 200 ${}^{\circ }$C or 250 ${}^{\circ }$C. More research into the influence of noble metal dopants and how they can be modeled is given in [79,80,81]. In Figure 3, the symbols depict measured data, while the solid lines are best-fit power-law curves [47,76].

Tangirala et al. [78] recently studied the influence of different dopants on the sensitivity of an SnO${}_{2}$ film with regard to its response towards CO, shown in Figure 3c. The influence of Cu, Pt, and Pd is analyzed with the additives being incorporated using chemical (**Chem**) and impregnation (**Impe**) methods with urea (**U**) or ammonia (**A**) as precipitating agents. The study shows that the optimal sensitivity is reached, when chemical methods with urea as precipitant are used for dopant incorporation, while Cu was found to provide the highest sensitivity. The urea precipitator forms a uniform and homogeneous particle distribution during synthesis, which is why this method leads to an increased sensitivity. Furthermore, Cu was shown as the additive which provides the most sensitive response because the reaction of the copper atom with adsorbed oxygen to form CuO was found to be optimal at the sensor operating temperature.

## 3. Modeling the SMO Sensor Structure

In order to fully understand the effects of fabrication, the reliability, and the operation of an SMO gas sensor, measurements alone are not sufficient. Modeling and simulations are of critical importance and serve as essential tools to allow for a thorough analysis into the interplay between different materials without expensive, complex, and time-consuming experiments. Simulations allow to gain a better understanding of its operation and long-term reliability. In this section we describe models used recently to simulate the CMOS-integrable fabrication of the SMO devices and electro-thermal models using the finite element method (FEM) for the analysis of the mechanical stability of the membrane structure. While the primary concern in the membrane structure is its mechanical stability, the microheater simulations are primarily geared towards power dissipation and temperature uniformity [82].

### 3.1. Fabrication

The requirement of a heater element is the primary source of the complexity in the design and fabrication of SMO sensors. The inclusion of a heater means that a suspended membrane must be produced, which houses a microheater and hosts the active sensor region. While several methods exist to create this membrane using MEMS techniques, performing this feat using CMOS techniques provides several advantages, mentioned in the previous section. Several means of fabrication can be used to produce the desired membrane. It can be formed by first depositing all the necessary layers, then etching from the back of the wafer, until the membrane is reached and thereby exposed. This back-side etching can be formed using deep reactive ion etching (DRIE). Alternatively, the membrane can be formed by etching from the top, through prepared openings in the membrane, which form suspended beams and the active region. This fabrication step can be formed using wet chemical or plasma etching. When etching proceeds from the wafer backside, a full or closed membrane is generated, resulting in thicker membranes (in the range of 1 μm to 2 μm) and a higher power dissipation compared to the suspended membrane alternatives. In addition, perfect alignment when etching from the back is difficult to achieve and can add to the cost of fabrication, since this method is not fully CMOS technology compatible [17,83]. When etching through pre-generated holes from the top of the wafer, a suspended membrane is achieved [35]. One advantage of this method is that the heat from the microheater can only escape through these thin suspension beams, resulting in a better power performance of the overall sensor.

**Figure 4:**
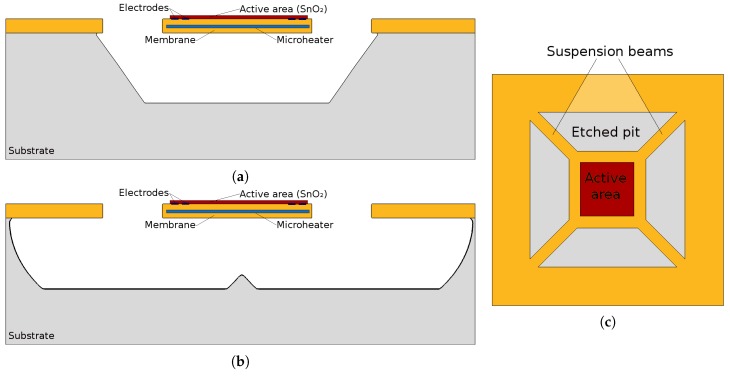
Suspended membrane of an SMO sensor device with a side view shown in (**a**,**b**) and a top view is given in (**c**). In (**a**,**b**) wet chemical etching using KOH and dry etching using SF${}_{6}$ plasma, respectively, are shown, with their differences clearly evident. In (**c**) the top view of the final suspended structure is shown.

In [84] we have performed a thorough analysis of the effects of fabrication of a tyical suspended membrane sensor with an active area of 100 μm×100 μm using both wet chemical etching with potassium hydroxide (KOH) and dry etching in sulfur hexafluoride (SF${}_{6}$) plasma. For a deeper understanding of the models used in the simulations, refer to [85] for KOH etching and [86,87] for SF${}_{6}$ plasma etching. The simulation software used is ViennaTS [88]. The etching simulations proceeded as follows:

- Wet chemical etching: After a 150 min KOH bath with a concentration of 30% at 70 ${}^{\circ }$C the right size of hole was generated and the suspended membrane was released. The etch rate for the silicon wafer depends on the crystallographic orientation which, under the processing setup used, was found to be 13.3 nm/s, 24.2 nm/s, 0.1 nm/s and 23.9 nm/s for directions <100>, <110>, <111>, and <311>, respectively. Although the final structure, shown in Figure 4a appears to be very smooth and clean, with no unwanted lateral etching, the wet chemical etching step can be very corrosive to FEOL devices and surrounding features. Therefore, as an additional alternative analysis using plasma etching was carried out for the same structure. Plasma etching is a commonly used process in CMOS fabrication, which is much less corrosive than a wet chemical bath.
- Dry plasma etching: The simulation for plasma etching involves a stochastic approach for particles which represent the molecules, atoms, and ions, all commonly found in a plasma etch chamber. While not all particles which are found in the chamber contribute to the etch rate, the ones which do are simulated using Monte Carlo ray tracing. The particles can be neutral or charged, representing the chemical and physical accelerated ion etch components, respectively. Because the physical etching component etches layers indiscriminately, the chemical etch is the main contributor, since it can be more selective. However, the negative aspect is the resulting lateral etch, since chemical etching is non-directional. In Figure 4b the increased amount of lateral etching is evident. This simulation setup involved an SF${}_{6}$ plasma chemistry with a surface fluorine flux of 1 × 10${}^{19}$ cm${}^{-1}$ s${}^{-1}$. The required plasma etch time was found to be much shorter than the wet chemical bath, as a 300 s etch was long enough to fully expose the membrane, as shown in Figure 4b.

In a follow-up analysis of the two structures, it was discovered that the lateral etching noted in the plasma-etched structure shows no adverse effects on the stress distribution in the active region of the sensor [84]. The stress after deposition is a combination of all the intrinsic and thermal stresses in the individual layers which together make up the membrane. Minimizing this stress is crucial for the overall structure’s stability. Typical values for the as-deposited stress, which is a sum of the intrinsic and thermal components, in the Si${}_{3}$N${}_{4}$ and SiO${}_{2}$ membrane layers are about 1 GPa tensile and about 300 MPa compressive stress, respectively. When layers are stacked, it is important to have the combined stresses remain below 100 MPa, which is why it is critical that some layers produce a tensile stress, while others a compressive one, as they can cancel each other out in a stacked structure. The two types of residual stresses are the intrinsic stress which builds up during the deposition process and is frequently modeled using Volmer-Weber growth [89], and thermal stress which arises due to the cooling to room temperature of the film after deposition at elevated temperatures. The thermal stress also arises during device operation, since elevated temperatures are required to initiate the surface chemisorption process [44].

The deposition of the sensing layer itself should also be compatible with CMOS fabrication. For this reason, many ways in which SnO${}_{2}$ can be deposited have been studied. This includes chemical vapor deposition, sputtering, pulsed layer deposition, sol-gel processes, rheotaxial growth and vacuum oxidation, and spray pyrolysis [47,76]. Sputtering and spray pyrolysis are methods which can be very straight-forwardly implemented as a post CMOS fabrication step and they are very cost effective. In [90] using a combination of experiments and 3D simulations, we found that an isotropic coverage can be expected at corners and around edges using spray pyrolysis. However, this comes at a cost of deposition at elevated temperature (400 ${}^{\circ }$C) resulting in a higher thermal residual stress. To minimize residual stresses, an appropriate set of process parameters must be found and the fabrication process must be well controlled. Mechanical properties such as density, stoichiometry, orientation, and the average grain size of each layer of the sensor are defined by the specific deposition conditions. For example, some mechanical characteristics of the sensor layers can be shifted by annealing for one or more cycles.

### 3.2. Mechanical Stability

One major challenge in designing an SMO gas sensor is having excellent thermo-mechanical stability. As mentioned in the previous section the stacked structure can suffer from thermally induced stresses during the fabrication stage, but also during operation. In order to model these stresses properly, one must analyze the internal stress accumulation in the sensor micro-hotplate and the membrane layers, including Si${}_{3}$N${}_{4}$ and SiO${}_{2}$. The stress can directly influence several aspects of the sensor including the generation of crystalline defects, adhesion between layers, and the formation of film surface growth. Another concern is stress build-up in very small regions, which must be understood and controlled as it can be the source of an initial crack or delamination, ultimately resulting in device failure [91]. The thermal stress ($\sigma_{th}$) contributes significantly to the stress build-up within the membrane layers and is modeled using

(2) $$\sigma_{th}=E\alpha \left(T-T_{0}\right),$$

where *E* is the Young modulus, α is the coefficient of thermal expansion, and $T-T_{0}$ is the temperature difference leading to the rise in stress. For bilateral combinations, such as a thin film on top of a substrate, the strain in the film and substrate are expressed using

(3) $$\varepsilon_{film}=\alpha_{film}\Delta T+\frac{F_{film}\left(1-v_{film}\right)}{E_{film}t_{film}w}$$

and

(4) $$\varepsilon_{sub}=\alpha_{sub}\Delta T+\frac{F_{film}\left(1-v_{sub}\right)}{E_{sub}t_{sub}w}\;\;,$$

where $F_{film}$ is the thermal mismatch force, *w* is the width, *v* is the Poisson’s ratio, and *t* is the thickness. When the strain of the film and substrate are identical ($\varepsilon_{film}=\varepsilon_{sub}$), then $F_{film}$ can be found by

(5) $$F_{film}=\frac{w\left(\alpha_{sub}\alpha_{film}\right)\Delta T}{\frac{1-v_{film}}{t_{film}E_{film}}+\frac{1-v_{sub}}{t_{sub}E_{sub}}}\;\;.$$

In the case that $t_{sub}E_{sub}-v_{sub}\gg t_{film}E_{film}-v_{film}$, the thermal stress can be expressed using

(6) $$\sigma_{film}\left(T\right)=\frac{F_{film}}{t_{film}w}=\frac{\left(\sigma_{sub}-\sigma_{film}\right)\Delta TE_{film}}{1-v_{film}}$$

### 3.3. Electro-Thermo-Mechanical Analysis

**Figure 5:**
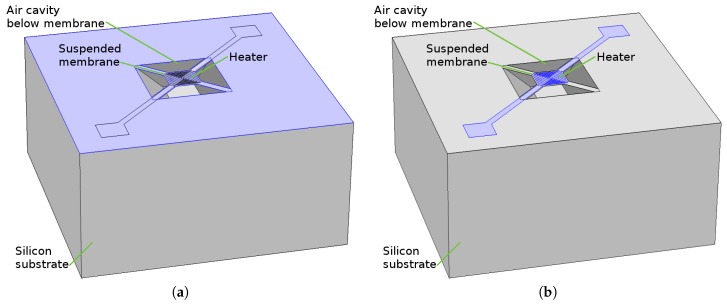
Simplified geometry of a device used in the electro-thermo-mechanical FEM model. In (**a**) the membrane materials are highlighted, which can include silicon nitride and silicon dioxide. In (**b**) the microheater is highlighted. The sensing layer and electrodes are not shown.

**Figure 6:**
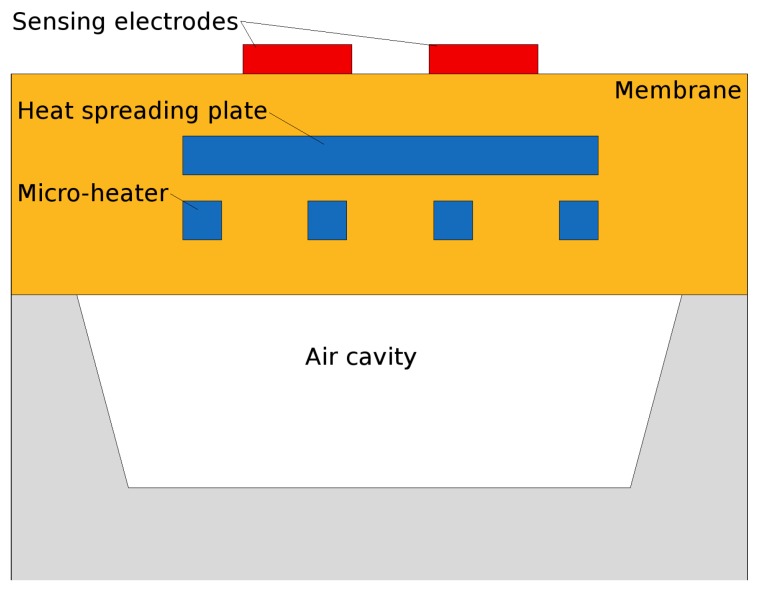
Schematic of the layers composing the membrane of the hotplate.

In order to perform a thorough electro-thermo-mechanical simulation of the desired structure, the finite element method (FEM) is applied. The geometry of the device is designed using a computer aided design (CAD) tool, such as SolidWorks and imported into a FEM simulation software, which can be COMSOL Multiphysics, ANSYS, ConventorWare, MEMS+, or IntelliSense. One simple example of a suspended membrane SMO sensor and micro-heater is shown in Figure 5 and Figure 6. In Figure 6 we also see the presence of a heat spreading plate which is commonly used in order to improve the temperature uniformity across the active region of the sensor [92]. The electrodes for gas sensing are placed on top of the membrane above the heat spreading plate. The heater can have a variety of shapes, including meander, curved, double spiral, elliptical, circular, plane plate, fin shape, honeycomb, or other irregular shapes [47,93,94]. The goal of the various designs is to ensure the highest temperature uniformity. The heater and other embedded layers are much thinner than the total membrane thickness, which is primarily SiO${}_{2}$ and Si${}_{3}$N${}_{4}$. In addition, the thicknesses of all layers are much smaller compared to their widths, making the aspect ratio a potential problem when modeling 3D structures. The large aspect ratio can cause the number of mesh elements to be unreasonably high, resulting in unfeasible memory requirements and unreasonable simulation times on commercial desktop computers. One way to manage these issues is to define an initial two-dimensional (2D) mesh in the plane of the membrane and to sweep it through the orthogonal direction. This method is effective when all stacked layers have the same lateral dimensions, but the sweep function becomes very complex when the layers have different shapes. An alternative is drawing the thin layers as two-dimensional objects and modeling them as shell elements, meaning that the vertical dimension is not explicitly simulated, but rather a linear extrapolation is used [92]. This solution simplifies the geometry and may result in some errors. Therefore, the properties of the shell elements must be calibrated carefully to avoid non-physical results.

When assigning material properties for the simulation, it is essential that these are calibrated to the fabrication process. Published values for many parameters can vary significantly, since they depend considerably on the fabrication process [92]. An example is the thermal conductivity which is strongly influenced by the quality of the crystal structure in the layer, which is in-turn defined by the fabrication process and post-fabrication annealing. Particularly the deposition temperature, growth speed, and other factors determining the distribution and size of the crystal grains have a major influence on the material [95]. The thickness of the layers can also change their properties, which is especially true for mechanical properties related to stress build-up, stress distribution, and mechanical stability. The operating temperature could potentially also influence the material properties, including the electrical and thermal conductivities and the Young modulus, which are all temperature-dependent. It is therefore essential to include all these dependences adequately in the simulation.

After the geometry, a mesh, and the material properties are defined, the physical behavior of the entire system must be simulated. The device is first biased by applying a potential across the circuit in order to have a current flowing through it, reaching the heater. The heater is a resistive element which converts the electrical power into heat due to Joule heating. The material and shape of the heater must be chosen so that the provided temperature is uniformly distributed across the active area while minimizing the power dissipation. The increase in temperature provided by Joule heating causes the sensing film to heat up. However, also the membrane materials increase in temperature as a consequence. This temperature increase and subsequent decrease, when the circuit is no longer biased, can bring up mechanical strain. The materials all have different coefficients of thermal expansion (CTE) causing mechanical deformation, when the device is biased, which could lead to eventual fracture or delamination.

#### 3.3.1. Electro-Thermal Behavior

**Figure 7:**
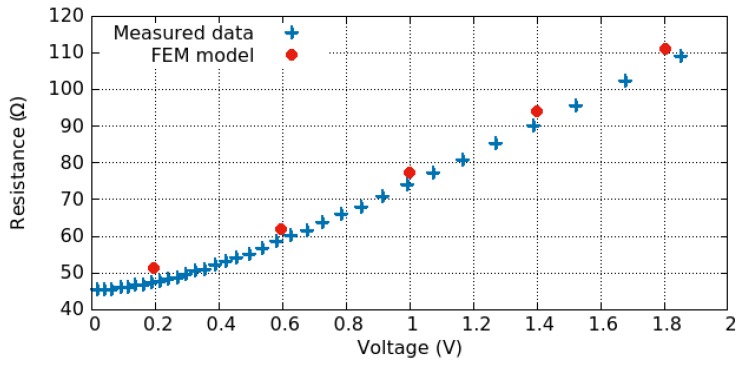
Resistance of the conductive layer of the sensor at different applied biases.

The Joule heating behavior is modeled using electro-thermal FEM simulations. First the electric behavior is described with

(7) $$\nabla \cdot \vec{j}=Q_{j,v}\;\;,$$

(8) $$\vec{j}=\sigma \vec{E}\;\;,$$

and

(9) $$\vec{E}=-\Delta V\;\;,$$

where $\vec{j}$ is the current density, $Q_{j,v}$ is the current source, σ is the electrical conductivity, $\vec{E}$ is the electric field, and *V* is the applied potential. When shell elements are used for approximation, the equations must be modified in order to make use of the tangential gradient operator which removes the normal component of the applied potential [92]. After the electrical behavior is modeled, the resistance is found with

(10) $$R=\frac{A}{\vec{j}}V\;\;,$$

where A is the cross-sectional area of the film. The relationship between the resistance and the applied voltage is plotted in Figure 7, where the described model reproduces the measured data very well [92].

After modeling the electrical behavior, the thermal problem can be modeled using the heat equation, which is defined over an entire structure with

(11) $$\nabla \cdot \vec{q}=-Q_{j}\;\;,$$

where $Q_{j}$ is the Joule heat and $\vec{q}$ is defined by

(12) $$\vec{q}=k\nabla T\;\;,$$

where *T* is the temperature. The Joule heat is defined by

(13) $$Q_{j}=\vec{j}\cdot \vec{E}\;\;.$$

**Figure 8:**
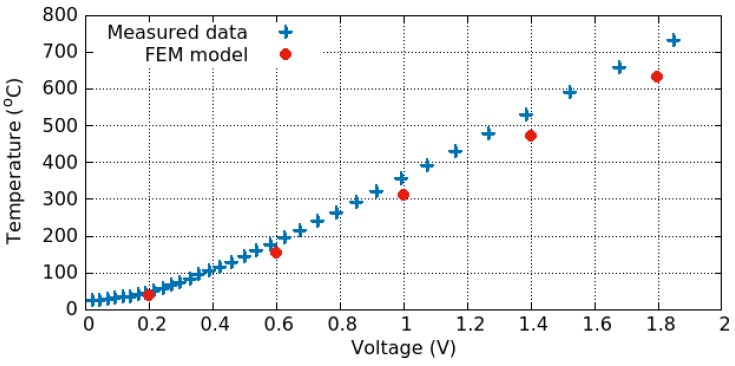
Temperature obtained using an FEM model compared with one calculated using the measured resistance.

**Figure 9:**
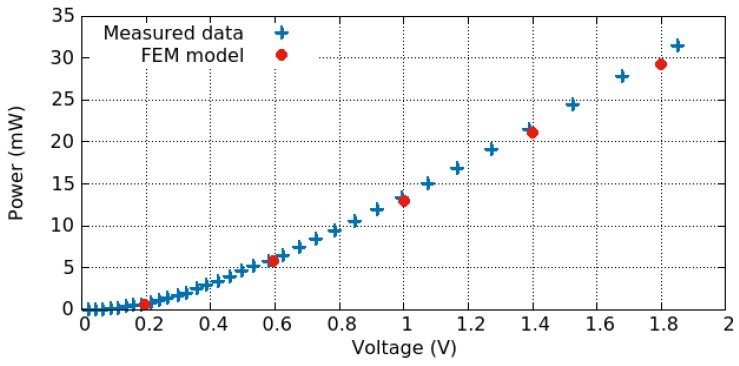
FEM simulation and measured power dissipated by the sensor for different applied biases.

The heat equation includes the conduction, but not convection of heat, which could take place in air. In many simulations the convection is neglected; however, at very high temperatures the convection should be included, which could be calculated using

(14) $$Q_{conv}=h\cdot A_{t}\cdot \Delta T\;\;,$$

where *h* is the mean heat transfer coefficient and $A_{t}$ is the exposed area from which the heat flows. The relationship between the temperature obtained on the hotplate and the applied voltage is plotted in Figure 8 and the relationship between the dissipated power and the applied potential is shown in Figure 9. The model reproduces the measured results quite well. The difference evidenced in the temperature plot could be attributed to the fact that the temperature cannot be measured or read directly. Rather, it is calculated based on several measurements of the resistance at different temperatures and empirical parameters determined through additional experiments using the formula [92]

(15) $$R=R_{25}\left[1+a\left(T-25\right)+b{\left(T-25\right)}^{2}\right],$$

where *R* is the measured resistance at a specific bias and $R_{25}$ is the resistance at 25 ${}^{\circ }$C, while *a* and *b* are empirical parameters determined through additional experiments.

Coppeta et al. [92] also looked at the influence of the humidity on the temperature profile of the hotplate. They found that the air thermal conductivity can diverge at different humidity levels, once the applied bias is above 2 V. However, this ultimately had little to no influence on the temperature obtained for different biases. At the highest tested applied bias (4.2 V) the air thermal conductivity showed a variation of 15%, when the humidity varied from 25% to 75%. In this range, the temperature varied by only 5 ${}^{\circ }$C, corresponding to about 4%.

#### 3.3.2. Thermo-Mechanical Behavior

The mechanical behavior of the sensor under operation is a critical component to ensure its long-term reliability and thereby long operational lifetime. The repeated heating and cooling of the membrane layers could potentially create high levels of deformation and bending leading to, in the worst case, cracking and complete device failure. The bending is related to the stress accumulated inside it due to the post-fabrication intrinsic stress, discussed earlier, and the thermal stress which arises due to the constant heating and cooling. Every material responds differently to changes in temperature and each of the membrane materials has a different coefficient of thermal expansion, shown in Table 2.

In order to appropriately model the thermo-mechanical behavior and to obtain the stress and displacement of the membrane films, the material parameters used in the FEM model must be calibrated. As is evident in Table 2 the material properties which play a large role in the stress build-up can vary for each material depending on the fabrication process. Material parameters such as the CTE, Young’s modulus, Poisson’s ratio, density, and thermal conductivity can vary somewhat, thereby influencing the sensor’s thermo-mechanical behavior [92].

Joule heating causes the deformation of the structure by increasing the temperature in the membrane layers by $\Delta T=\left(T-T_{0}\right)$, resulting in a thermal strain ($\varepsilon_{th}$) of

(16) $$\varepsilon_{th}=\alpha \cdot \Delta T\;\;,$$

where α is the coefficient of thermal expansion. Assuming that the materials behave linearly elastic, they are described by Hooke’s law, relating the elastic strain $\varepsilon_{el}$ and stress *S* with

(17) $$S=S_{0}+C:\varepsilon_{th}\;\;,$$

where *C* is the stiffness tensor which depends on the Young’s modulus and the Poisson ratio in the case of an isotropic body. $S_{0}$ is the intrinsic stress in the layer. This calculation is performed on all FEM elements at all indices *ij* with

(18) $$\sigma_{ij}=S_{0,ij}+\sum_{k=1}^{3}\sum_{l=1}^{3}c_{ij,kl}\varepsilon_{kl}\;\;.$$

The displacement vector ($\vec{u}$) resulting from the induced strain is found by solving

(19) $$\varepsilon_{th}=\frac{1}{2}\left[{\left(\nabla \vec{u}\right)}^{T}+\nabla \vec{u}\right].$$

In order to perform the displacement simulation, at least one point in the simulation space must be fixed. Otherwise, instead of a displacement, the entire structure would shift. For FEM simulations, the bottom surface of the silicon domain is fixed. This way, the calculation gives a displacement with respect to the bulk wafer.

**Figure 10:**
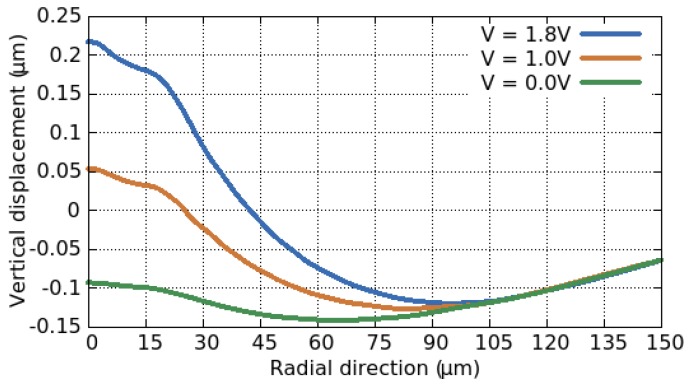
Simulation results of the out-of-plane displacement of the top surface of the membrane along its radius. The hotplate is biased and the deformation is caused by the intrinsic and the thermal stress caused by the temperature increase due to the Joule effect.

FEM simulations on a typical SMO sensor geometry were performed in [92] with the displacement along the membrane from the middle (radial direction = 0 μm) outward shown in Figure 10. The membrane tends to bend downward, when no bias is applied due to the intrinsic stresses. The center of the membrane, which hosts the heater and heat spreading plate and serves as the active region, has an intrinsic-stress induced displacement of −100 nm. When the bias is increased to 1.8 V, the displacement in the central area increases to 200 nm out of plane, a difference of 300 nm. Furthermore, without bias applied, the stress along the suspension beams and in the membrane is relatively even throughout. However, when a bias is applied and Joule heating is taking place, the stress tends to concentrate around the heated section of the membrane and the suspension beams serve to dampen the stress from its maximum value (where the beams meet the suspended membrane) to its minimum value (where the suspension beams are connected to the wafer).

## 4. Modeling the Thermal Response

The choice of microheater and membrane design are critical to achieve a uniform temperature distribution across the active region of the sensor. The temperature determines the quality of the sensing mechanism and is therefore one of the most important aspects, which should be carefully controlled during operation. Materials with high thermal conductivity are necessary to achieve the desired temperature distribution. However, using high thermally conducting films can increase thermal leakage to the silicon substrate, resulting in a high power loss. Recently, many researchers have increased their efforts to find the right material for the microheater and the right geometry to improve the temperature uniformity. One solution, proposed by Lahlalia et al. in [16], is to use a dual-hotplate design which introduces a single circular microheater along with two passive micro-hotplates. The operating principle of this design is enabled by the high thermal conductivity of the microheater layer compared to the membrane materials. Often, in order to understand the relationship between the applied bias, the resulting heater temperature and consumed power, and to ultimately improve the sensor, many simulations must be performed, until an optimized design is found. For this purpose, the FEM is not always desired, due to the stringent numerical requirements for complex geometrical designs and the need for transient simulations, which can increase the simulation time to an unreasonable scale. An alternate solution is using an equivalent electric circuit to discretize the microheater and the membrane in a Cauer model.

### 4.1. Heater Materials

The microheater is a critical component of SMO gas sensors, as it provides the temperature required for the sensing mechanism to be initiated. The area where the sensing layer is deposited is the active region or active area. This region must have a precise and uniform temperature distribution across it in order to ensure predictable and reliable sensor operation. The exact temperature depends on the target gases and the sensitive materials used. Therefore, the choice of microheater material plays a key role in determining the sensor performance and long-term reliability [76,96]. The microheater material should have a low thermal conductivity, high electrical resistivity, high melting point, low thermal expansion coefficient, low Poisson’s ratio, and low fabrication cost which can be achieved through integration with CMOS fabrication technologies [35]. At the early stages of microheater development, the commonly used microheater materials were aluminum or gold, as these were also readily used in standard integrated circuit designs [97,98]. Later, it was shown that these materials suffer from oxide formation at high temperatures, low resistivity, poor contact properties, and electro-migration effects [35]. With this in mind, researchers started using platinum which is today frequently applied to heating elements for temperatures below 500 ${}^{\circ }$C. Platinum allows for high current densities, is chemically inert, and has a stable temperature coefficient of resistance (TCR) up to 650 ${}^{\circ }$C [99]. However, this material is expensive and making an electrical contact between it and other materials is not straightforward. Furthermore, platinum has a positive TCR, which magnifies the effect of hotspots, potentially impacting long-term reliability.

Researchers are currently looking for new materials to overcome the limitations of the previously mentioned options. More recently, nickel and iron-nickel have been used for microheaters. Their low TCR and thermal conductivity make them suitable candidates [100,101]. Materials like tungsten [102,103], nickel-chromium alloys [104], Dilver P1 [105], molybdenum [106], hafnium diboride [107], titanium nitride [108], silicon carbide [109], and Sb-doped SnO${}_{2}$ [110] have also found to have potential for the heating element owing to the several positive features, namely, low thermal expansion, resistance to humidity, a high Young’s modulus, and their non-magnetic nature.

Tungsten was reported by Ali et al. [111] as a good high temperature heater material, while Lahlalia et al. [17] looked into tantalum-aluminum (TaAl), which is characterized by its ability to retain its mechanical strength at high temperatures. TaAl also has a negative TCR of about −100 ppm/${}^{\circ }$C, leading to minimal hotspot formations and a stable temperature versus input power. Ultimately, when choosing a microheater material, it is not a one-material-fits-all exercise, but rather the material choice depends considerably on the desired requirements. However, it is very clear that the microheater has a big influence on the operation, stability, and long term reliability of the SMO gas sensor. Sensitivity, selectivity, and response time are partially dependent on the thermal behavior of the heating element. The desirable features of a microheater are low power consumption, temperature stability, and temperature uniformity over the entire sensitive layer. One method used in an attempt to improve the microheater operation without changing the material is through creative microheater geometries. However, many complex geometries will result in an increased stress in the microheater, so this must be kept in mind, when new designs are presented. A high stress in any thin layer of the sensor can result in an unreliable performance and a reduced lifetime of the entire device. Furthermore, current crowding in thinned lines and corners of the microheater are another factor which must be taken into account. Many engineers use rounded corners and circular heater structures in order to mitigate current crowding which may lead to the generation of microcracks, localized deformations, and various electro-migration phenomena [92].

### 4.2. Heater Designs

**Figure 11:**
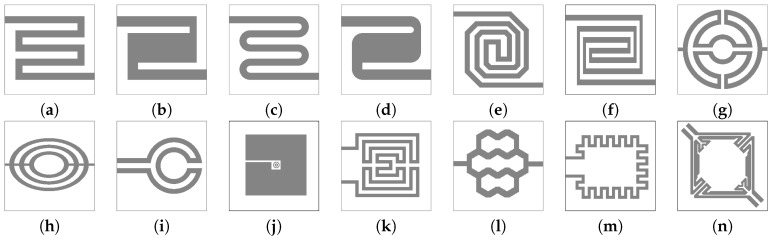
Microheater geometries characterized and modeled over the last decades. The shapes depicted are: (**a**) Meander, (**b**) S-meander, (**c**) Curved, (**d**) S-curved, (**e**) Double spiral, (**f**) Square double spiral, (**g**) Drive wheel, (**h**) Elliptical, (**i**) Circular, (**j**) Plane plate, (**k**) Fin shape, (**l**) Honeycomb, (**m**) Loop shape, (**n**) Irregular.

The design of the microheater is an additional way in which the temperature distribution across the active area can be controlled. To improve the temperature uniformity and reduce power consumption, many designs have been attempted, shown in Figure 11. Several researchers introduce a highly thermally conductive plate above or below the microheater, which is electrically inert, to distribute the heat more evenly [92], as shown in Figure 6. This added plate introduces a further photolithography step, increasing the cost of fabrication. Another option is to add more metal to existing geometries, which distributes heat, but has relatively little influence on the power dissipation, as was done in [16] with the dual-hotplate design. There, the authors introduced two hotplates inside of the circular design, resulting in very good temperature uniformity at 300 ${}^{\circ }$C at a very low power consumption of 8 mW.

From the designs shown in Figure 11, some variations of the the meander and circular geometries are most commonly used for gas sensors. Several recent works study the influence of the geometry, membrane design, and choice of heater material on the performance and stability of the gas sensor [16,17,34,92]. The studies have all shown that a reduction in the membrane thickness results in a reduced power consumption. This is the primary reason why suspended membranes are desired over a full membrane, as heat can only escape through the suspension beams and the thinner those are, the better the power performance of the device. In [34] the authors directly compared a meander and circular design with a combination of experiment and finite element simulations. The meander design had a broader suspension beam width which directly caused an increase in power consumption. However, this design also provided a faster temperature response since increasing the power by one mW resulted in an increase in temperature of 7 ${}^{\circ }$C, compared to 4 ${}^{\circ }$C for the design with narrower suspension beams. Therefore, the choice membrane and beam thicknesses, as well as the heater design will depend strongly on the device requirements and trade-offs must be made, since improvements in one aspect (e.g., power consumption) will likely lead to a reduced performance in another (e.g., mechanical stability).

Furthermore, the influence of the microheater on the sensing behavior has recently been reported in [112]. When slight openings were introduced in the microheater, the temperature uniformity, mechanical stability, and the sensitivity of the gas sensor improved at no cost to the power consumption. Recently, the efficiency of published microheaters was characterized by evaluating the efficiency which is defined as the increase in temperature over a 1 mm${}^{2}$ area when 1 mW is applied to the microheater (mm${}^{2}\cdot$K/mW). While many other studies describe efficiency in terms of ${}^{\circ }$C/mW, this is insufficient when attempting to characterize the quality of microheaters over a wide range of sizes, which was our goal, since the size of the heaters significanctly influences the power consumption of the entire sensor [47]. A trend was extracted form this that larger microheaters generally have better power efficiency, supported by the fact that several researchers characterized two heaters which were designed using identical processes, but where the surface area varied. The results showed that a larger heater predictably requires more power, but shows an improved power efficiency, when compared to a smaller one [103,113]. This finding gives us more motivation to design microheater arrays instead of gas sensor arrays with individual microheatears. One such array was proposed recently, which provides 270 ${}^{\circ }$C and 350 ${}^{\circ }$C simultaneously with a very fast response (40 μs) and a low power consumption (9.31 mW) [16].

### 4.3. Heat Loss Mechanisms

**Figure 12:**
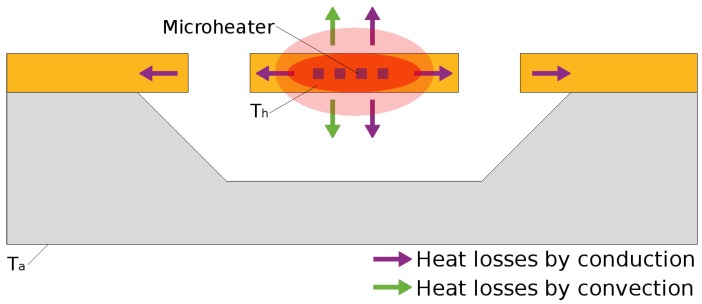
Heat loss mechanisms through the SMO gas sensor, where T${}_{h}$ and T${}_{a}$ correspond to the temperature of the microheater and the ambient temperature. The figure shows the locations of the principal heat losses of conduction, convection, and radiation which is represented by red ellipses.

For an SMO sensor, three mechanisms must simultaneously be taken into consideration when modeling heat losses and power consumption: conduction, convection, and radiation. In general, radiation is ignored for temperatures below 600 ${}^{\circ }$C as it is insignificant compared to conduction and convection at lower temperatures. Most of the heat losses are caused by heat conduction through the micro-hotplate and the air as well as heat convection through the heat exchange between the external faces of the membrane and the surrounding air, as depicted in Figure 12.

#### 4.3.1. Conduction

Heat conduction is the heat transfer from the heated area of the membrane to the substrate, through the suspension beams [35]. To simplify the model, the heat conduction perpendicular to the membrane is neglected due to the small thickness of the layers which compose the membrane stack and only the lateral flow is simulated. Therefore, a one-dimensional heat conduction problem in cuboid coordinates is often sufficient to model this behavior. For a suspended membrane with four suspension beams through which heat can flow, the heat losses by conduction are expressed by

(20) $$Q_{cond}=\frac{4\cdot \lambda_{T}\cdot A_{beam}\left(T_{h}-T_{a}\right)}{l}\;\;,$$

where $\lambda_{T}$ is the thermal conductivity of the membrane stack and $A_{beam}$ and *l* are the sectional area and length of each beam. Because the membrane is not a single material, but rather a stack with varying thermal conductivities, $\lambda_{T}$ for the entire stack is calculated using

(21) $$\lambda_{T}=\sum_{k=1}^{n}\lambda_{k}\times \frac{t_{k}}{\sum_{k=1}^{n}t_{k}}\;\;,$$

where $t_{k}$ is the thickness of each layer *k*, $\lambda_{k}$ is the thermal conductivity of layer *k*, and *n* is the total number of stacked layers.

#### 4.3.2. Convection

Convection is the heat transfer between the heated surface and the surrounding fluid which includes air and other gases [114]. This mechanism is described by a combination of fluid motion and heat conduction through air. Fluid motion occurs, when differences in temperature cause the air to move from heated to cool sections. The calculation of heat losses by natural convection $Q_{conv}$ is expressed by Newton’s law of cooling as

(22) $$Q_{conv}=h\cdot A\left(T_{h}-T_{a}\right),$$

where *h* is the mean heat transfer coefficient and *A* is the exposed area from which the heat flows.

#### 4.3.3. Radiation

The heat transfer which takes the form of electromagnetic waves in the infrared region is referred to as radiation. Radiation is emitted by a body due to the thermal agitation of its composing molecules [92,115]. Assuming that the heated membrane area behaves like a grey body, the heat losses by radiation are expressed using

(23) $$Q_{rad}=\epsilon \cdot \sigma_{S}\cdot A\left(T_{h}^{4}-T_{a}^{4}\right),$$

where ϵ is the emissivity, $\sigma_{S}$ is the Stefan constant (5.67 × 10${}^{-8}$ Wm${}^{-2}$ K${}^{-4}$), and *A* is the surface area of the object emitting the radiation. While radiation is ignored for lower temperatures, it is quite clear that, due to the $T^{4}$ dependence, it can have a significant impact, when the microheater is operating at high temperature.

### 4.4. Transient Response

The thermal transient response refers to the time required to heat the active sensor from the ambient temperature, usually room temperature, $T_{a}$ to the target temperature $T_{h}$ which can be several hundreds degrees Celsius. In principle, by ignoring the geometry of the sensor and the thermal distribution inside the structure, the thermal response can be modeled using the simple equation

(24) $$C_{th}\frac{\partial T\left(t\right)}{\partial t}=\frac{T_{h}-T_{a}}{R_{th}}+P_{in}\;\;,$$

where $C_{th}$ is the thermal capacitance of the micro-hotplate, $R_{th}$ is the thermal resistance, and $P_{in}$ is the input power. The equation can be solved using Fourier and Laplace analysis with the boundary conditions $T_{t=0}=T_{a}$ and $T_{t=\infty }=T_{h}$. The temperature of the heated element observes the exponential behavior

(25) $$T\left(t\right)=\left(T_{h}-T_{a}\right)e^{-t/\tau }+T_{a}\;\;,$$

where $\tau =R_{th}C_{th}$ is the thermal time constant. Accordingly, a micro-hotplate having a small thermal resistance and low thermal mass will give a faster response. Therefore, a small heater exhibits a faster thermal response thanks to the smaller heat capacity, allowing for operation with very short pulse times, reducing the power dissipation drastically. This is the primary reason why scaling and miniaturization is highly sought after.

In order to simulate and verify the thermal performance of the sensor, we must analyze the temperature distribution, thermal response time, temperature gradient, heat losses, and heat exchange between the sensor and its environment. Simulating all these phenomena within a FEM environment would be quite challenging, especially if very small heaters are being simulated, meaning that temperature changes occur quite quickly. Therefore, additional TCAD tools to complement the FEM are indispensable to analyze the in-depth thermal behavior of devices. These simulations can be performed by first discretizing the entire membrane structure of the sensor with a fine mesh. For each of the mesh elements or at the nodes, the temperature influence over time can be calculated, according to Equations (24) and (25). Ultimately, this approach would lead to the finite-difference method which also requires solutions to be found at every node of a complex mesh. A further option is to extract a lumped thermal network, where the equations can be solved analytically using standard circuit analysis and converting the thermal parameters by their thermal equivalents, as shown in Table 3. This method based on the Cauer network model [17,116,117] is useful when trying to optimize the microheater geometry without having to execute long and complex FEM simulations.

**Figure 13:**
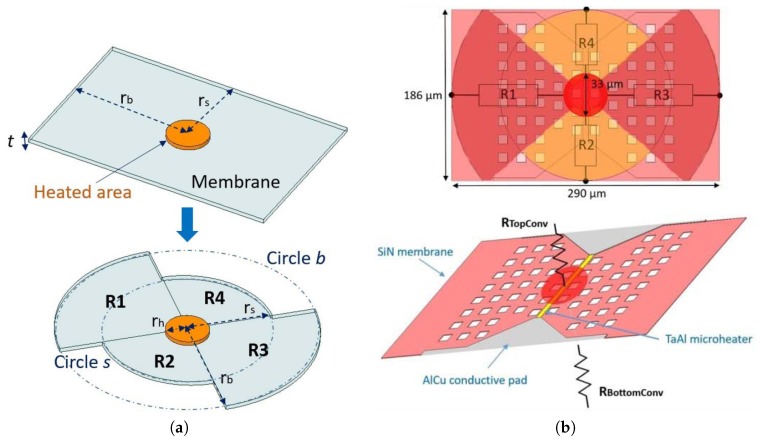
Sensor geometry from [17] used to test the Cauer network model. In (**a**) the geometry used when considering heat loss by conduction is shown, while in (**b**) the treatment of the heat loss mechanism by convection is depicted on the bottom and the membrane size and extracted thermal resistances are shown on top.

This method of using an electrical circuit to represent the thermal behavior of an SMO sensor was presented for a complex microheater structure in [17] and in [16]. The authors therein showed that with some calibration, the method is very powerful and can accurately model the thermal behavior of a complex SMO structure. The structure modeled is shown in Figure 13, where the geometries and extracted thermal resistors used in the Cauer network are shown. For conduction, shown in Figure 13a, the rectangular membrane is replaced by two circular bow-tie membranes, which are easier to calculate as they allow for a one-dimensional treatment of the heat problem using cylindrical coordinates. Four thermal resistors are sufficient to represent the full membrane. For convection, shown in Figure 13b, first the mean heat transfer coefficient is calculated, as described in [17] and in [16]. The convecting resistances from the top and bottom of the membrane are shown, which are placed in parallel to the thermal resistors from Figure 13a as part of the model.

**Figure 14:**
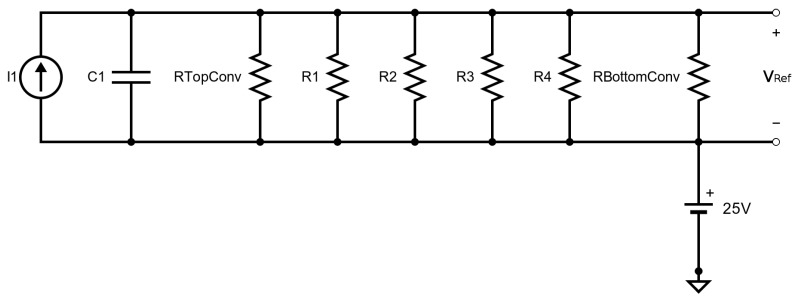
Cauer model reproduction of a microheater design presented in [17], which is further used to analyze the transient behavior of the heater.

The resistances calculated from Figure 13 are placed in parallel in an electrical circuit, shown in Figure 14. As can be observed from Table 3 the capacitor C1 corresponds to the thermal capacitance, the +25 V source is to reflect a room temperature of 25 ${}^{\circ }$C, the I1 current source corresponds to the applied power, while reading out V${}_{\mathrm{Ref}}$ gives the achieved temperature, in Celsius. The thermal resistances (R1, R2, R3, and R4) are calculated using

(26) $$R_{th}=\frac{t}{k\cdot A}\;\;,$$

where *t* is the thickness of the layer, *k* is the thermal conductivity, and *A* is the cross-sectional area in the normal direction to the heat flow. Likewise, the thermal capacitance is calculated using

(27) $$C_{th}=c\cdot \rho \cdot V\;\;,$$

where *c* is the specific heat, ρ is the material density, and *V* is the volume of the material. The convection resistances $R_{\mathrm{TopConv}}$ and $R_{\mathrm{BottomConv}}$ are calculated using

(28) $$R_{conv}=\frac{1}{h\cdot A_{r}}\;\;,$$

where *h* is the heat transfer coefficient to air and $A_{r}$ is the exposed area from which the heat flows.

**Figure 15:**
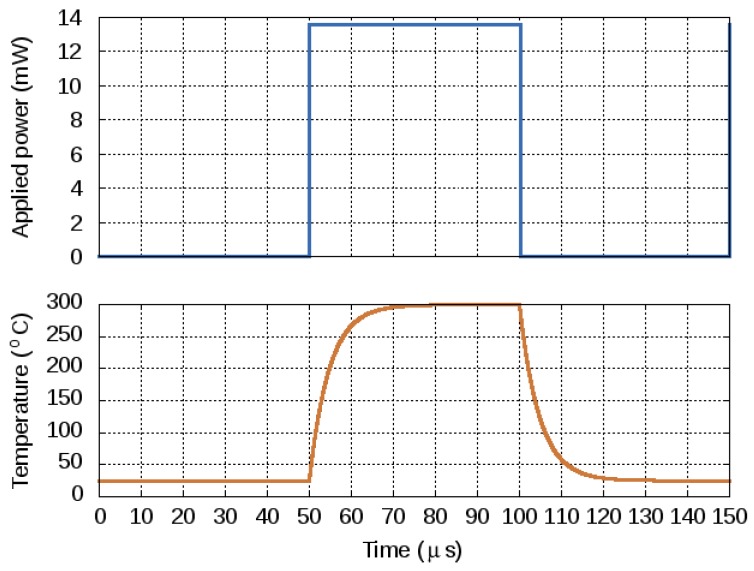
Transient response of the reference temperature (potential V${}_{\mathrm{Ref}}$) when a perfect square signal with an amplitude of 13.55 mW is applied at the power (current I1) source from Figure 14.

To model the electrical circuit, a current source is added. As can be seen in Table 3 an electrical current source corresponds to the heat flow rate in watts in its thermal parameter equivalent. It was found that, for the sensor presented in [17] an applied heat flow or thermal power (current) of 13.55 mW (mA) was sufficient to reach a target temperature (voltage) of 300 ${}^{\circ }$C (V). Analyzing the voltage evolution as a function of the current allows to estimate the power consumption of the gas sensor at a variety of temperatures. The transient response, based on the Cauer network model is shown in Figure 15. The heated area reached 300 ${}^{\circ }$C within about 20 μs and it takes about as much time to cool back to room temperature.

**Figure 16:**
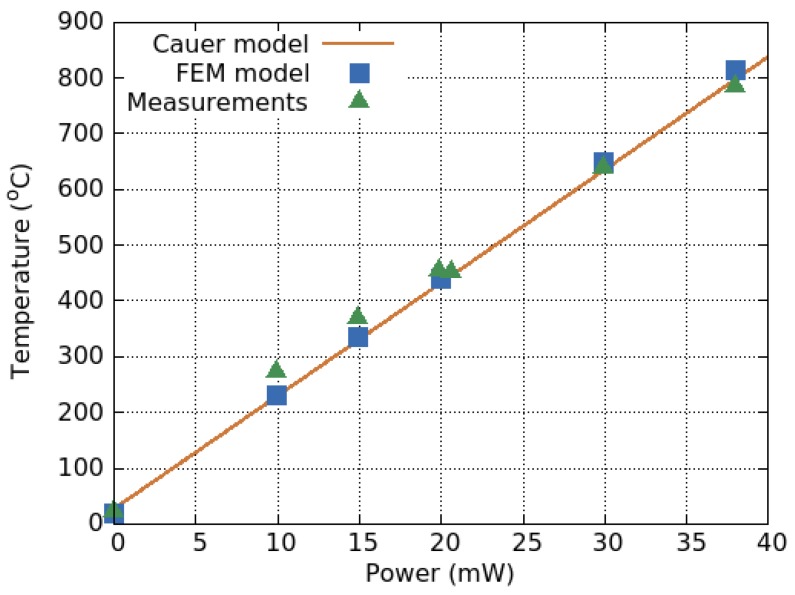
Experimental and simulated results for the temperature versus applied power relationship for a microheater design described in [17]. It is evident that the analytical Cauer model can reproduce the temperature-power relationship of an SMO sensor.

The presented model was compared to FEM simulations and measured data, with the results shown in Figure 16. Therein, we note that both the FEM model and the analytical model can reproduce the measured behavior. For the experimental values, the authors used resistive platinum and chromium silicon (CrSi) layers on top of the microheater in order to measure the generated temperature. A more complex microheater and membrane structure was modeled using the same method in [16]. The membrane structure was composed of four arms and a curved geometry was used in place of sharp edges and corners to reduce electron and heat accumulation therein. A representative electrical circuit required twenty-four (24) thermal resistors and two convection resistors (top and bottom). For that design, 6.3 mW was required to heat the sensing area to 300 ${}^{\circ }$C and the results once again found good agreement with the finite element simulation.

## 5. Modeling the SMO Sensing Mechanism

Considerable progress has recently been made towards understanding the conducting mechanism of SMO gas sensors and the surface adsorption reactions between the SMO film’s surface and the ambient gas molecules. In this section we describe the conductivity model for SMO films as well as the most recent research into the surface reactions taking place, how they modify the conductivity of the film, and how these effects can be modeled.

### 5.1. SMO Conductivity

The mechanism of gas sensing for an SMO film is related to a changing conductivity due to the presence of a surface charge distribution, brought upon by gas molecule adsorption. Therefore, the conductivity is the primary parameter which must be addressed during modeling. The electrical conduction of SMO films, just like many other semiconductors, can be described using drift diffusion equations [118] given by

(29) $$\nabla \cdot \vec{j_{n}}=R\;\;,$$

(30) $$\nabla \cdot \vec{j_{p}}=-R\;\;,$$

(31) $$\vec{j_{n}}=qn\mu_{n}\vec{E}+qD_{n}\nabla n\;\;,$$

(32) $$\vec{j_{p}}=qp\mu_{p}\vec{E}+qD_{p}\nabla p\;\;,$$

where $\vec{j_{x}}$ is the current density, *R* is the recombination rate, $\mu_{x}$ is the mobility of the majority carriers, $D_{x}$ is the diffusion constant for the majority carriers, *q* is the charge of an electron, and $\vec{E}=-\nabla V$ is the applied electric field. The values of *n* and *p* refer to the electron and hole concentrations in an n-type or p-type semiconductor, respectively. The symbol ${}_{x}$ refers to ${}_{n}$ or ${}_{p}$ where they represent the n-type or p-type semiconductor with electrons or holes as majority carriers, respectively. The electric field is calculated by solving the Poisson equation, which relates the electrical potential to the surface charge density ρ and the permittivity $\epsilon_{0}\epsilon_{r}$ with

(33) $$-\nabla \cdot \left(\varepsilon_{0}\varepsilon_{r}\nabla V\left(\vec{x}\right)\right)=\rho \;\;,$$

where $\vec{x}$ refers to the simulation space, since the potential is a gradient. The boundary conditions are Dirichlet at the ohmic contacts, since the potential is known there, and otherwise, the Neumann boundary condition is set. For an n-type semiconductor (e.g., SnO${}_{2}$), only (29) and (31) are considered, while (30) and (32) are calculated for a p-type semiconductor (e.g., CuO). It is also generally assumed that the thickness of the depletion layer, caused by the charges added by chemisorption at the surface during sensing, is close in size to the electron mean free path. Therefore, the diffusion terms can be removed from (31) and (32), which greatly simplifies the problem. The remainder of our discussion will concentrate on calculating n-type semiconductors using the concentration *n* and mobility $\mu_{n}$ to calculate the conductivity $\sigma =\vec{j_{n}}/\vec{E}$ with

(34) $$\sigma =q\cdot n\cdot \mu_{n}\;\;.$$

**Figure 17:**
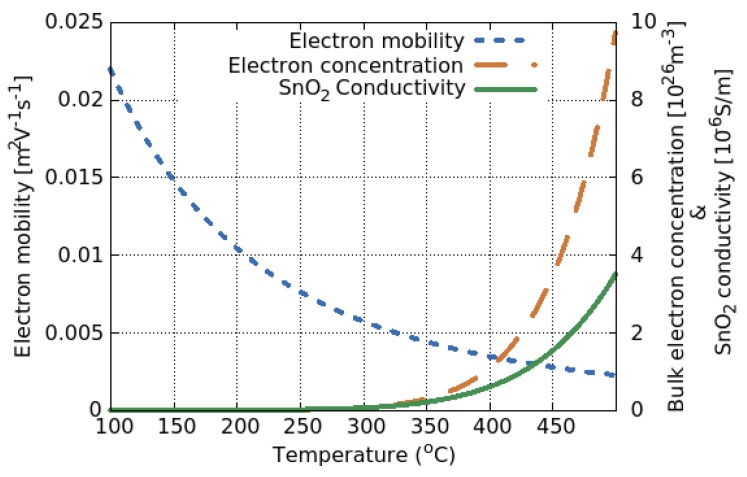
The influence of temperature on the electron mobility (μ), the electron concentration (*n*), and the SnO${}_{2}$ conductivity.

Even in an inert ambient, a change in temperature can influence the conductivity through a change in both the concentration and mobility of the majority carriers [119], shown in Figure 17.

The mobility of the charge carriers changes with temperature, but it remains unchanged during sensing through gas molecule adsorption. Therefore, the sensing mechanism depends on the conductive change in the SMO layer, caused by a change in the charge concentration [79]. During adsorption, the gas molecule will either donate an electron or it will take one from the SMO film’s bulk. By assuming fully ionized donors, the Schottky approximation is valid and the surface electron concentration is calculated using the Boltzmann distribution with

(35) $$n_{s}=N_{D}\cdot e^{-qV_{s}/k_{b}T}\;,$$

where $N_{D}$ is the donor density, $qV_{s}$ is the energy of the resulting bending in the surface band, $k_{B}$ is the Boltzmann constant, and *T* is temperature. From (35) we note that the conductance—and therefore also resistance—is a function of surface band bending. When modeling this behavior, the surface charges are used to find the resulting surface potential with

(36) $$V_{s}=-\frac{q\cdot N_{eff}^{2}}{2\varepsilon_{0}\varepsilon_{r}N_{D}}\;\;,$$

where $N_{eff}$ is the sum of all electrons which are donated by the adsorped molecule and those with enough energy to reach the surface from the bulk. Therefore, when the surface adsorption is modeled, the primary goal is to obtain $N_{eff}$ since this allows for the calculation of the surface potential, the resulting electric field, the depth of the depletion or accumulation region, and ultimately the resulting change in the film’s conductivity.

### 5.2. Surface Reactions

**Figure 18:**
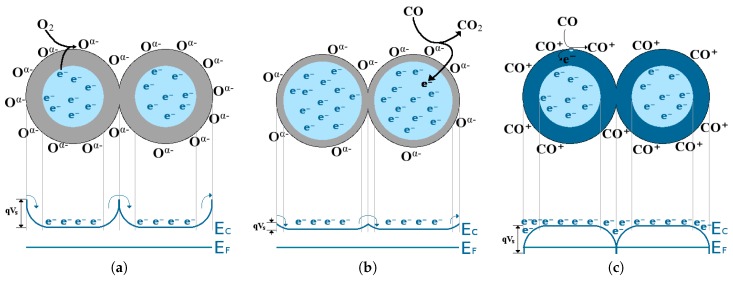
Gas sensing and resulting band bending for a granular film. (**a**) O${}^{\alpha -}$ adsorbs on the surface, creating a depletion region and band bending, (**b**) CO reacts with oxygen, thinning the depletion region and reducing band bending, and (**c**) CO adsorbs directly on the surface, creating an accumulation region and band bending.

A typical example commonly used to describe the surface reactions taking place during gas sensing with SMO films is that of carbon monoxide (CO) sensing, or the adsorption of a CO molecule on the surface of e.g., SnO${}_{2}$. This mechanism has been researched extensively and all the steps thought to be taking place during adsorption are described in [120]. The basic summary is that the gas sensing mechanism proceeds in the presence of oxygen gas and that oxygen ionosorbs on the surface in O${}^{-}$ or O${}^{2-}$ forms, thereby taking one or two electrons from the bulk, respectively, and creating a depletion region. This depletion region results in band bending, as shown in Figure 18.

There are several cases of gas adsorption on the surface of an SMO film. While they are all described by surface reduction and re-oxidation by oxygen, there are notable differences. The following situations are given in [79,121] to describe these various phenomena:

1. In an inert environment (e.g., N${}_{2}$) the surface energy bands are flat and no depletion or accumulation region is present. The number of charges at the surface is the same as that found in the bulk.
2. When oxygen is present in the environment, the oxygen vacancies found on the surface of the SMO film are filled by adsorbed O${}^{-}$ or O${}^{2-}$ and one or two charges are trapped, respectively. The bulk donates one or two electrons to the adsorbed oxygen and a depletion region is formed, resulting in energy band bending, depicted in Figure 18a.
3. When CO gas is in the ambient together with O${}_{2}$ gas the oxygen will be adsorped on the surface and subsequently removed by CO to form CO${}_{2}$. The surface will, thereby continuously re-oxidize, leading to a reduction in the depletion region, which depends on the amount of CO found. This is depicted in Figure 18b.
4. If only a target gas is present without any oxygen, an oxygen vacancy at the surface of the SMO film can react with a CO molecule, ultimately reducing the surface. In this interaction, CO donates an electron on the surface, forming an accumulation region, depicted in Figure 18c.

#### 5.2.1. Mass Action Law

To model the surface reactions, the set of reactions taking place are usually simplified to include the dominant reactions, including the adsorpion of oxygens species with

(37) $$\frac{1}{2}O_{2,gas}+S+\alpha \cdot e^{-}⇌O_{S}^{\alpha -}\;\;,$$

α=1 or α=2 in the case of strongly or doubly ionized oxygen, respectively, *S* is a surface adsorption site, $O_{S}^{\alpha -}$ refers to a chemisorbed oxygen species, and $e^{-}$ is an electron in the conduction band. The electron exchange with the tin oxide Sn is given by

(38) $$H_{2}O^{gas}+2\cdot Sn_{Sn}+O_{S}^{\alpha -}⇌2\cdot \left(SnOH\right)+\alpha \cdot e^{-}\;\;,$$

where $Sn_{Sn}$ is a surface tin atom. In the presence of a reducing gas such as CO, the final reaction taking place is

(39) $$CO^{gas}+O_{S}^{\alpha -}\to CO_{2}^{gas}+\alpha \cdot e^{-}+S\;\;.$$

From the above reactions, a rate equation for the oxygen surface coverage $\left[O_{S}^{\alpha -}\right]$ in the steady state can be obtained using the mass action law

(40) $$\begin{array}{cc} \frac{d\left[O_{S}^{\alpha -}\right]}{dt}= & k_{ads}\cdot p_{O_{2}}^{0.5}\cdot \left[S\right]\cdot n_{S}^{\alpha }-k_{des}\cdot \left[O_{S}^{\alpha -}\right]-k_{react}\cdot \left[O_{S}^{\alpha -}\right]\cdot p_{CO}\\ & -k_{H_{2}O,ads}\cdot p_{H_{2}O}\cdot {\left[Sn_{Sn}\right]}^{2}\cdot \left[O_{S}^{\alpha -}\right]+k_{H_{2}O,des}\cdot {\left[OH\right]}^{2}\cdot e\cdot n_{S}^{\alpha }\;\;, \end{array}$$

where $k_{ads/des}$ and $k_{H_{2}O,ads/H_{2}O,des}$ refer to the reaction constants for oxygen and water vapor adsorption/desorption, respectively and $k_{react}$ is the reaction constant for the reaction of CO with adsorbed oxygen. The square brackets [ ] denote surface coverage, $n_{S}$ is the surface concentration of electrons, and $p_{O_{2}/CO}$ are partial pressures of $O_{2}$/CO, respectively. The calculated value of $n_{S}$ is added to $n_{s}$ from (35) to obtain $N_{eff}$ from (36) in order to model the final change in surface potential and calculate the induced change in conductivity. This model has been successfully applied to simulate a sensor with a porous SnO${}_{2}$ film [58] as well as SnO${}_{2}$ nanowires [118].

#### 5.2.2. Power Law Response

From several experiments it has been observed that the change in conduction due to exposure to a target gas follows a power law response [76,122]. With this in mind, Hua et al. [122,123,124] developed a model to simulate the change in SnO${}_{2}$ conductivity after it is exposed to CO in dry air, thereby avoiding any influence of ambient humidity. The transducer model assumes two conducting mechanisms taking place, described by the Schottky barrier model and the grain model. The receptor functions are modeled using the mass action law as described above. The derived model for the power-law exponent *n* in the presence of species *A* is given by

(41) $$n=\frac{\alpha }{2\cdot \beta }\left(1-\frac{1}{\beta \cdot m^{2}+1}\right),$$

where α=1 for dissociative or α=2 for non-dissociative adsorption, as discussed before, and β is the charge state of oxygen which has adsorbed on the surface. The parameter *m* refers to the reduced surface charge density and is a fraction which describes the number of donors over the charge density per unit length of the depletion region.

#### 5.2.3. Langmuir Adsorption Model

Recent studies also show that the adsorption mechanism of formaldehyde (CH${}_{2}$O) and ethanol (C${}_{2}$H${}_{5}$OH) on SnO${}_{2}$ with platinum nanoparticles can be described using the Langmuir isotherm model [112,125]. The authors consider the overall resistance of the sensing film as *i* resistances *R* in parallel, where each *R* is composed of *k* resistances *r* in series. The parameters *R*, *r*, and *i* model the resistance of a single layer of the film, a single site on that layer, and the number of conduction paths, respectively, while *k* represents the number of active sites in a single conductive monolayer. The resistance is then modeled using

(42) $$R=k\cdot \beta \cdot r_{1}+k\left(1-\beta \right)r_{0}\;\;,$$

where $r_{0}$ and $r_{1}$ are the resistances of a vacant and occupied surface site, respectively, and β is the surface adsorption coverage. Following the Langmuir adsorption model, β is expressed with

(43) $$\beta =\frac{T_{k}C}{1+T_{k}\cdot C}\;\;,$$

where $T_{k}$ is the adsorption equilibrium constant and *C* is the concentration of the analyte. The sensing response can then be derived by combining the above expressions to obtain

(44) $$S=\frac{k}{i}\left(r_{1}-r_{0}\right)\left(\frac{T_{k}\cdot C}{1+T_{k}\cdot C}\right).$$

**Figure 19:**
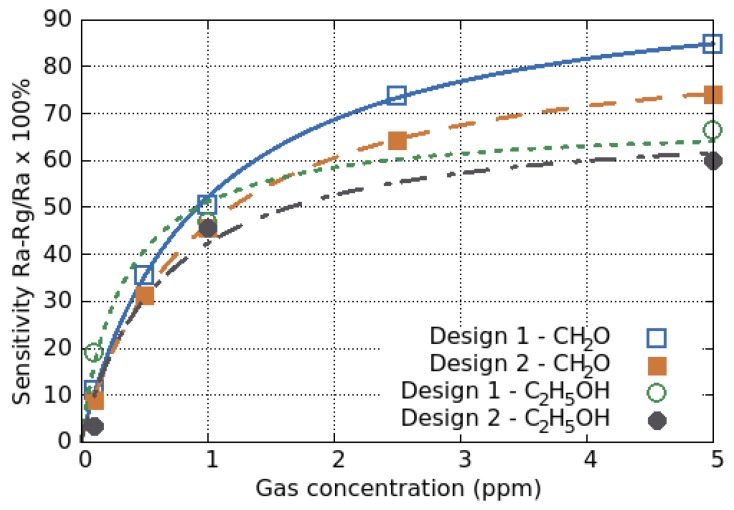
Sensing response of two different designs towards exposure to formaldehyde (CH${}_{2}$O) and ethanol (C${}_{2}$H${}_{5}$OH) concentrations up to 5 ppm on an SnO${}_{2}$ film with Pt nanoparticles. The symbols are measured results and lines are best fits lines using the Langmuir adsorption model.

The model is then simplified to

(45) $$S=a\frac{b\cdot C}{1+b\cdot C}\;\;,$$

where *a* and *b* are fitting parameters which represent the change in resistance for an occupied site and the adsorption equilibrium constant, respectively. This model was used to successfully model the behavior of CH${}_{2}$O and C${}_{2}$H${}_{5}$OH adsorption on platinum-doped SnO${}_{2}$, as shown in Figure 19. Therein, the symbols represent the experimental measurements, performed at 0.1 ppm, 0.5 ppm, 1 ppm, 2.5 ppm, and 5 ppm concentrations of the desired gas, while the lines show the fit to the Langmuir model from (45) [112].

## 6. Conclusions

We offer an overview of the most important aspects in modeling and simulation of semiconductor metal oxide gas sensors, concentrating on the micro-hotplate and membrane materials. After introducing different concepts for gas sensing, we present the case for the SMO sensor as the one which has the most potential for integration with CMOS fabrication and electrical integrated circuits. By enabling this integration, one particular goal of the gas sensor industry, to enable sensor inclusion in portable and wearable devices, can be achieved. In order to ensure sufficient progress in gas sensor design and to enable miniaturization and power dissipation down to levels appropriate for portable and wearable devices, new materials and designs must be studied, calibrated, and examined for their reliability and operational stability. It is inefficient to try out different designs and calibrate sensors using experiments only. Modeling and simulation play an essential role for improving the next generation of gas sensors. Simulations and TCAD have revolutionized the microelectronics industry and the advancements achieved over the last decades are owed to the tremendous achievements made in modeling the physical behavior of integrated circuits down to their individual components and material make-up.

In order to apply the same idea to SMO gas sensors, the FEM plays the most prominent role. In this manuscript we showed recent successful attempts to model the electro-thermo-mechanical behavior of gas sensors and the models which make up its primary feature, the microheater and membrane which hosts it. First, we presented the models used to generate (or release) the membrane in order to create an air cavity beneath it. For this purpose, a couple of models are discussed, including plasma etching using SF${}_{6}$ plasma and wet KOH etching. Both of these methods can be worked into a CMOS fabrication sequence and they both have their advantages and disadvantages. However, the stress build-up during film deposition is one of the principal concerns for fabrication. The intrinsic stress build-up during deposition was examined, including the residual component which arises due to the manner in which granular structures is deposited, and the thermal component which arises when a high-temperature deposition process is followed by cooling to room temperature. This stress component must be included in the subsequent electro-thermo-mechanical FEM model in order to ensure a physically accurate simulation.

The electro-thermal behavior of the microheater was modeled using Joule heating within a FEM environment. The temperature reached by the heater as a function of the applied bias was simulated and compared to measurements showing a very good match. The same is the case with the relationship between the power dissipation and the voltage. The heating of the membrane layers can result in strain build-up which causes deformation and can lead to cracking or delamination failures. A model for the thermo-mechanical behavior of the sensor was also presented and the importance of using proper material properties was highlighted. The main material properties of interest include the CTE, Young’s modulus, Poisson’s ratio, density, and thermal conductivity, all of which vary to some degree depending on the means of fabrication and the post-deposition annealing steps.

An alternative to the FEM was presented to model the heat loss mechanisms involved in heating the microheater. This involved building a Cauer network, which was modeled in an integrated circuit simulation environment and where each electrical parameter corresponds to a thermal equivalent. This method can be used to quickly model the electro-thermal behavior of microheaters, which would otherwise be too cumbersome when a fine mesh is required, as is the case for FEM simulations. A Cauer network for a sample microheater was given, which was composed of six resistors (four thermal conducting resistors and two convective resistors) one capacitor (thermal capacitor), a power source (current source), and a battery, where the potential represents the temperature reached. This simple representation was able to reproduce the plot of temperature versus applied power compared to measurements and the FEM model, while taking seconds to simulate instead of hours or days. Ultimately modeling and simulation are an integral part of design and the ability to simulate the physical behavior of SMO sensors brings us one step closer to creating designs which can be fully integrated into portable electronics and wearables.

Finally, recent achievements made in understanding and modeling the mechanisms of SMO films’ operation is provided. While the conductivity of the sensing film is modeled using drift diffusion equations, the surface reactions taking place are modeled with the mass action law. It has been long thought that an oxygen rich environment is essential in order to induce sensing through the initial adsorption of oxygen on the surface, which would take an electron from the SMO bulk, thereby increasing the resistance and creating a band bending at the interface. The subsequent exposure to a target gas would see that gas’s molecule react with the adsorbed oxygen, returning the electron to the SMO film and in the process reducing the resistivity and the band bending effect. We show the applicability of the mass action law, power law, and Langmuir adsorption model to mathematically model this sensing behavior presented by several recent works based on an SnO${}_{2}$ sensing film in the presence of carbon monoxide, formaldehyde, and ethanol. However, other studies have shown that even in the absence of oxygen, some gas molecules adsorb on the surface vacancy sites of the SMO film, donating an electron and creating an accumulation region. This behavior needs further studies before it can be included in a general model for SMO sensing. The development of a simulation tool for SMO sensors would ultimately be a great benefit to sensor designers in order to design new structures and optimize them outside of the cost-intensive laboratory environment.

## Figures and Tables

**Table 1:** Summary of available gas sensing technologies and their evaluation according to several parameters, including sensitivity, accuracy, selectivity, response time, stability, durability, power, cost, and footprint. The number ratings correspond to: 4 = Excellent, 3 = Good, 2 = Fair, 1 = Poor.

Parameter	SMO	Catalyic Pellistor	Piezo- Electric	Electro- Chemical	Thermal Pellistor	Photo- Ionization	Infrared Adsorption
Sensitivity	4	3	4	3	1	4	4
Accuracy	3	3	4	3	3	4	4
Selectivity	2	1	2	3	1	2	4
Response time	4	3	4	2	3	4	2
Stability	3	3	3	1	3	4	3
Durability	3	3	2	2	3	4	4
Power	4	4	2	3	3	1	2
Cost	4	4	3	3	3	2	2
Footprint	4	3	3	2	3	4	1

**Table 2:** Material properties ranges for layers typically found in the SMO sensor membrane.

Material	CTE (10${}^{-6}$ K${}^{-1}$)	Young’s Modulus (GPa)	Poisson’S Ratio	Density (Mg/m${}^{3}$)	Thermal Conductivity (W·m${}^{-1}\cdot$K${}^{-1}$)
SiO${}_{2}$	0.55–0.75	66.3–74.8	0.15–0.19	2.17–2.65	1.3–1.5
Si${}_{3}$N${}_{4}$	1.4–3.7	166–297	0.23–0.28	2.37–3.25	10–43
Si	7–8	140–180	0.265–0.275	2.28–2.38	84–100
Aluminum	16–24	68–88.5	0.32–0.36	2.57–2.95	80–220
Platinum	8.8–9.2	154–172	0.385–0.395	21.45–21.47	70–72
Tungsten	4.2–4.6	340–410	0.27–0.29	19.25–19.35	170–175

**Table 3:** Thermal to electrical parametric conversion for lumped thermal network simulations using the Cauer model.

Thermal Parameter	Electrical Equivalent
Temperature (K)	Voltage (V)
Specific heat (J kg${}^{-1}$ K${}^{-1}$)	Permittivity (F m${}^{-1}$)
Thermal resistivity (K m W${}^{-1}$)	Electrical resistivity (Ω m)
Thermal resistance (K W${}^{-1}$)	Resistance (V A${}^{-1}$)
Heat flow (W)	Current (A)
Heat (J = W s)	Charge (C = A s)
Thermal conductivity (W K${}^{-1}$ m${}^{-1}$)	Electrical conductivity (S m${}^{-1}$)
Capacitance (J K${}^{-1}$)	Capacitance (F)

## Abbreviations

The following abbreviations are used in this manuscript:

2D	two-dimensional
3D	three-dimensional
ADC	analog-to-digital converter
BEOL	back end of line
CAD	computer aided design
CMOS	complementary metal oxide semiconductor
CTE	coefficient of thermal expansion
CVD	chemical vapor deposition
DRIE	deep reactive ion etching
FEM	finite element method
FEOL	front end of line
KOH	potassium hydroxide
IoE	internet of everything
IoT	internet of things
IR	infrared
ITO	indium tin oxide
MEMS	micro-electro-mechanical systems
RC	resistance/capacitance
RF	radio frequency
RAM	random access memory
ROM	read-only memory
SMO	semiconductor metal oxide
TCAD	technology computer aided design
TCR	temperature coefficient of resistance

## References

[1] Martinelli G., Carotta M.C., Ferroni M., Sadaoka Y., Traversa E. (1999). Screen-printed perovskite-type thick films as gas sensors for environmental monitoring. Sens. Actuators B Chem..

[2] Carotta M.C., Ferroni M., Gnani D., Guidi V., Merli M., Martinelli G., Casale M., Notaro M. (1999). Nanostructured pure and Nb-doped TiO_2_ as thick film gas sensors for environmental monitoring. Sens. Actuators B Chem..

[3] Fine G.F., Cavanagh L.M., Afonja A., Binions R. (2010). Metal oxide semi-conductor gas sensors in environmental monitoring. Sensors.

[4] Li H., Mu X., Yang Y., Mason A.J. (2014). Low power multimode electrochemical gas sensor array system for wearable health and safety monitoring. IEEE Sens. J..

[5] Moos R., Müller R., Plog C., Knezevic A., Leye H., Irion E., Braun T., Marquardt K.J., Binder K. (2002). Selective ammonia exhaust gas sensor for automotive applications. Sens. Actuators B Chem..

[6] Riegel J., Neumann H., Wiedenmann H.M. (2002). Exhaust gas sensors for automotive emission control. Solid State Ion..

[7] Wales D.J., Grand J., Ting V.P., Burke R.D., Edler K.J., Bowen C.R., Mintova S., Burrows A.D. (2015). Gas sensing using porous materials for automotive applications. Chem. Soc. Rev..

[8] Wang F., Gu H., Swager T.M. (2008). Carbon nanotube/polythiophene chemiresistive sensors for chemical warfare agents. J. Am. Chem. Soc..

[9] Tomchenko A.A., Harmer G.P., Marquis B.T. (2005). Detection of chemical warfare agents using nanostructured metal oxide sensors. Sens. Actuators B Chem..

[10] Yoo R., Cho S., Song M.J., Lee W. (2015). Highly sensitive gas sensor based on Al-doped ZnO nanoparticles for detection of dimethyl methylphosphonate as a chemical warfare agent simulant. Sens. Actuators B Chem..

[11] Moore G.E. (1965). Cramming more components onto integrated circuits. Electronics.

[12] Arden W., Brillouët M., Cogez P., Graef M., Huizing B., Mahnkopf R. (2010). More-Than-Moore White Paper. http://itrs2.net/uploads/4/9/7/7/49775221/irc-itrs-mtm-v2_3.pdf.

[13] Zhang G.Q., van Roosmalen A. (2010). More Than Moore: Creating High Value Micro/Nanoelectronics Systems.

[14] Tiggelaar R.M., Sanders R.G., Groenland A., Gardeniers J.G. (2009). Stability of thin platinum films implemented in high-temperature microdevices. Sens. Actuators A Phys..

[15] Tommasi A., Cocuzza M., Perrone D., Pirri C., Mosca R., Villani M., Delmonte N., Zappettini A., Calestani D., Marasso S. (2017). Modeling, fabrication and testing of a customizable micromachined hotplate for sensor applications. Sensors.

[16] Lahlalia A., Filipovic L., Selberherr S. (2018). Modeling and simulation of novel semiconducting metal oxide gas sensors for wearable devices. IEEE Sens. J..

[17] Lahlalia A., Le Neel O., Shankar R., Kam S.Y., Filipovic L. (2018). Electro-thermal simulation & characterization of a microheater for SMO gas sensors. J. Microelectromech. Syst..

[18] Maeder T., Sagalowicz L., Muralt P. (1998). Stabilized platinum electrodes for ferroelectric film deposition using Ti, Ta and Zr adhesion layers. Jpn. J. Appl. Phys..

[19] Johnson R.G., Holmen J.O., Foster R.B., Sridhar U. (1990). Adhesion Layer for Platinum Based Sensors. U.S. Patent.

[20] Klemenschits X., Selberherr S., Filipovic L. (2018). Modeling of gate stack patterning for advanced technology nodes: A review. Micromachines.

[21] Jin Z., Zhou H.J., Jin Z.L., Savinell R.F., Liu C.C. (1998). Application of nano-crystalline porous tin oxide thin film for CO sensing. Sens. Actuators B Chem..

[22] Ryu H.W., Park B.S., Akbar S.A., Lee W.S., Hong K.J., Seo Y.J., Shin D.C., Park J.S., Choi G.P. (2003). ZnO sol–gel derived porous film for CO gas sensing. Sens. Actuators B Chem..

[23] Zeng J., Hu M., Wang W., Chen H., Qin Y. (2012). NO_2_-sensing properties of porous WO_3_ gas sensor based on anodized sputtered tungsten thin film. Sens. Actuators B Chem..

[24] Mutinati G., Brunet E., Steinhauer S., Köck A., Teva J., Kraft J., Siegert J., Schrank F., Bertagnolli E. (2012). CMOS-integrable ultrathin SnO_2_ layer for smart gas sensor devices. Procedia Eng..

[25] Wang L., Wang S., Xu M., Hu X., Zhang H., Wang Y., Huang W. (2013). A Au-functionalized ZnO nanowire gas sensor for detection of benzene and toluene. Phys. Chem. Chem. Phys..

[26] Park S., Sun G.J., Jin C., Kim H.W., Lee S., Lee C. (2016). Synergistic effects of a combination of Cr_2_O_3_-functionalization and UV-irradiation techniques on the ethanol gas sensing performance of ZnO nanorod gas sensors. ACS Appl. Mater. Interfaces.

[27] Kozhushner M.A., Trakhtenberg L.I., Landerville A.C., Oleynik I.I. (2013). Theory of sensing response of nanostructured tin-dioxide thin films to reducing hydrogen gas. J. Phys. Chem. C.

[28] Liu X., Cheng B., Hu J., Qin H., Jiang M. (2008). Semiconducting gas sensor for ethanol based on LaMg_x_Fe_1-x_O_3_ nanocrystals. Sens. Actuators B Chem..

[29] Moon J., Park J.A., Lee S.J., Zyung T., Kim I.D. (2010). Pd-doped TiO_2_ nanofiber networks for gas sensor applications. Sens. Actuators B Chem..

[30] Zhou X., Liu J., Wang C., Sun P., Hu X., Li X., Shimanoe K., Yamazoe N., Lu G. (2015). Highly sensitive acetone gas sensor based on porous ZnFe_2_O_4_ nanospheres. Sens. Actuators B Chem..

[31] Chen J., Xu L., Li W., Gou X. (2005). *α*-Fe_2_O_3_ nanotubes in gas sensor and lithium-ion battery applications. Adv. Mater..

[32] Jing Z., Zhan J. (2008). Fabrication and gas-sensing properties of porous ZnO nanoplates. Adv. Mater..

[33] Neri G., Bonavita A., Micali G., Rizzo G., Callone E., Carturan G. (2008). Resistive CO gas sensors based on In_2_O_3_ and InSnO_x_ nanopowders synthesized via starch-aided sol–gel process for automotive applications. Sens. Actuators B Chem..

[34] Walden P., Kneer J., Knobelspies S., Kronast W., Mescheder U., Palzer S. (2015). Micromachined hotplate platform for the investigation of ink-jet printed, functionalized metal oxide nanoparticles. J. Microelectromech. Syst..

[35] Simon I., Bârsan N., Bauer M., Weimar U. (2001). Micromachined metal oxide gas sensors: Opportunities to improve sensor performance. Sens. Actuators B Chem..

[36] Frietsch M., Zudock F., Goschnick J., Bruns M. (2000). CuO catalytic membrane as selectivity trimmer for metal oxide gas sensors. Sens. Actuators B Chem..

[37] Annanouch F., Gràcia I., Figueras E., Llobet E., Cané C., Vallejos S. (2015). Localized aerosol-assisted CVD of nanomaterials for the fabrication of monolithic gas sensor microarrays. Sens. Actuators B Chem..

[38] Roy S., Basu S. (2002). Improved zinc oxide film for gas sensor applications. Bull. Mater. Sci..

[39] Filipovic L., Selberherr S., Mutinati G.C., Brunet E., Steinhauer S., Köck A., Teva J., Kraft J., Siegert J., Schrank F. (2014). Modeling the growth of tin dioxide using spray pyrolysis deposition for gas sensor applications. IEEE Trans. Semicond. Manuf..

[40] Brunet E., Maier T., Mutinati G., Steinhauer S., Köck A., Gspan C., Grogger W. (2012). Comparison of the gas sensing performance of SnO_2_ thin film and SnO_2_ nanowire sensors. Sens. Actuators B Chem..

[41] Neri G. (2015). First fifty years of chemoresistive gas sensors. Chemosensors.

[42] Qu Z., Fu Y., Yu B., Deng P., Xing L., Xue X. (2016). High and fast H_2_S response of NiO/ZnO nanowire nanogenerator as a self-powered gas sensor. Sens. Actuators B Chem..

[43] Singh E., Meyyappan M., Nalwa H.S. (2017). Flexible graphene-based wearable gas and chemical sensors. ACS Appl. Mater. Interfaces.

[44] Eranna G., Joshi B., Runthala D., Gupta R. (2004). Oxide materials for development of integrated gas sensors—A comprehensive review. Crit. Rev. Solid State Mater. Sci..

[45] Korotcenkov G. (2007). Metal oxides for solid-state gas sensors: What determines our choice?. Mater. Sci. Eng. B.

[46] Dey A. (2018). Semiconductor metal oxide gas sensors: A review. Mater. Sci. Eng. B.

[47] Filipovic L., Lahlalia A. (2018). System-on-chip SMO gas sensor integration in advanced CMOS technology. J. Electrochem. Soc..

[48] Liu X., Cheng S., Liu H., Hu S., Zhang D., Ning H. (2012). A survey on gas sensing technology. Sensors.

[49] Ponzoni A., Baratto C., Cattabiani N., Falasconi M., Galstyan V., Nunez-Carmona E., Rigoni F., Sberveglieri V., Zambotti G., Zappa D. (2017). Metal oxide gas sensors, a survey of selectivity issues addressed at the SENSOR Lab, Brescia (Italy). Sensors.

[50] Marquis B.T., Vetelino J.F. (2001). A semiconducting metal oxide sensor array for the detection of NO_x_ and NH_3_. Sens. Actuators B Chem..

[51] Mo Y., Okawa Y., Tajima M., Nakai T., Yoshiike N., Natukawa K. (2001). Micro-machined gas sensor array based on metal film micro-heater. Sens. Actuators B Chem..

[52] Barsan N., Koziej D., Weimar U. (2007). Metal oxide-based gas sensor research: How to?. Sens. Actuators B Chem..

[53] Ng K.T., Boussaid F., Bermak A. (2011). A CMOS single-chip gas recognition circuit for metal oxide gas sensor arrays. IEEE Trans. Circ. Syst. I Regul. Pap..

[54] Konduru T., Rains G.C., Li C. (2015). A customized metal oxide semiconductor-based gas sensor array for onion quality evaluation: System development and characterization. Sensors.

[55] Joshi N., Hayasaka T., Liu Y., Liu H., Oliveira O.N., Lin L. (2018). A review on chemiresistive room temperature gas sensors based on metal oxide nanostructures, graphene and 2D transition metal dichalcogenides. Microchim. Acta.

[56] Zhang D., Liu J., Jiang C., Liu A., Xia B. (2017). Quantitative detection of formaldehyde and ammonia gas via metal oxide-modified graphene-based sensor array combining with neural network model. Sens. Actuators B Chem..

[57] Woo Lee S., ping Tsai P., Chen H. (1997). H_2_ sensing behavior of MOCVD-derived SnO_2_ thin films. Sens. Actuators B Chem..

[58] Barsan N., Rebholz J., Weimar U. (2015). Conduction mechanism switch for SnO_2_ based sensors during operation in application relevant conditions; implications for modeling of sensing. Sens. Actuators B Chem..

[59] Korotcenkov G., Brinzari V., Pronin I., Ham M., Cho B. (2017). Metal oxides for application in conductometric gas sensors: How to choose. Solid State Phenomena.

[60] Gardner J.W., Guha P.K., Udrea F., Covington J.A. (2010). CMOS interfacing for integrated gas sensors: A review. IEEE Sens. J..

[61] Suh J.H., Cho I., Kang K., Kweon S.J., Lee M., Yoo H.J., Park I. (2018). Fully integrated and portable semiconductor-type multi-gas sensing module for IoT applications. Sens. Actuators B Chem..

[62] Brattain W.H., Bardeen J. (1953). Surface properties of germanium. Bell Syst. Tech. J..

[63] Seiyama T., Kato A., Fujiishi K., Nagatani M. (1962). A new detector for gaseous components using semiconductive thin films. Anal. Chem..

[64] Shaver P.J. (1967). Activated tungsten oxide gas detectors. Appl. Phys. Lett..

[65] ams AG (2016). Ultra-Low Power Analog VOC Sensor for Indoor Air Quality Monitoring, CCS801:v1-02. https://ams.com/documents/20143/36005/CCS801_DS000457_3-00.pdf.

[66] ams AG (2015). Air Quality Sensor, AS-MLV-P2:v1-01. https://ams.com/documents/20143/36005/AS-MLV-P2_DS000359_1-00.pdf.

[67] ams AG (2019). Ultra-Low Power Digital Gas Sensor for Monitoring Indoor Air Quality, CCS811:v1-00. https://ams.com/documents/20143/36005/CCS811_DS000459_7-00.pdf.

[68] Figaro USA, Inc. (2013). TGS 2600—For the Detection of Air Contaminants. http://www.figarosensor.com/product/entry/tgs2600.html.

[69] Figaro USA, Inc. (2015). TGS 2602—For the Detection of Air Contaminants. http://www.figarosensor.com/product/entry/tgs2602.html.

[70] Figaro USA, Inc. (2014). TGS 8100—For the Detection of Air Contaminants. http://www.figarosensor.com/product/entry/tgs8100.html.

[71] Taguchi N. (1972). Gas-Detecting Device. U.S. Patent.

[72] Taguchi N. (1971). Gas-detecting device. U.S. Patent.

[73] Velmathi G., Mohan S., Henry R. (2016). Analysis and review of tin oxide-based chemoresistive gas sensor. IETE Tech. Rev..

[74] Lackner E., Krainer J., Wimmer-Teubenbacher R., Sosada F., Deluca M., Gspan C., Rohracher K., Wachmann E., Köck A. (2017). Carbon monoxide detection with CMOS integrated thin film SnO_2_ gas sensor. Mater. Today Proc..

[75] Wimmer-Teubenbacher R., Steinhauer S., von Sicard O., Magori E., Siegert J., Rohracher K., Gspan C., Grogger W., Köck A. (2015). Gas sensing characterisation of CMOS integrated nanocrystalline SnO_2_–Au thin films. Mater. Today Proc..

[76] Filipovic L., Selberherr S. (2015). Performance and stress analysis of metal oxide films for CMOS-integrated gas sensors. Sensors.

[77] Mädler L., Sahm T., Gurlo A., Grunwaldt J.D., Barsan N., Weimar U., Pratsinis S.E. (2006). Sensing low concentrations of CO using flame-spray-made Pt/SnO_2_ nanoparticles. J. Nanopart. Res..

[78] Tangirala V., Gómez-Pozos H., Rodríguez-Lugo V., Olvera M. (2017). A study of the CO sensing responses of Cu-, Pt- and Pd-activated SnO_2_ sensors: Effect of precipitation agents, dopants and doping methods. Sensors.

[79] Degler D. (2017). Spectroscopic Insights in the Gas Detection Mechanism of tin Dioxide Based Gas Sensors. Ph.D. Thesis.

[80] Degler D., Müller S.A., Doronkin D.E., Wang D., Grunwaldt J.D., Weimar U., Barsan N. (2018). Platinum loaded tin dioxide: A model system for unravelling the interplay between heterogeneous catalysis and gas sensing. J. Mater. Chem. A.

[81] Müller S.A., Degler D., Feldmann C., Türk M., Moos R., Fink K., Studt F., Gerthsen D., Bârsan N., Grunwaldt J.D. (2018). Exploiting synergies in catalysis and gas sensing using noble metal-loaded oxide composites. ChemCatChem.

[82] Filipovic L., Selberherr S. (2016). Stress considerations for system-on-chip gas sensor integration in CMOS technology. IEEE Trans. Device Mater. Reliab..

[83] Elmi I., Zampolli S., Cozzani E., Mancarella F., Cardinali G. (2008). Development of ultra-low-power consumption MOX sensors with ppb-level VOC detection capabilities for emerging applications. Sens. Actuators B Chem..

[84] Filipovic L., Selberherr S. Processing of integrated gas sensor devices. Proceedings of the TENCON—2015 IEEE Region 10 Conference.

[85] Radjenović B., Radmilović-Radjenović M. (2009). 3D simulations of the profile evolution during anisotropic wet etching of silicon. Thin Solid Film..

[86] Gomez S., Jun Belen R., Kiehlbauch M., Aydil E.S. (2004). Etching of high aspect ratio structures in Si using SF_6_/O_2_ plasma. J. Vac. Sci. Technol. A Vac. Surf. Film..

[87] Belen R.J., Gomez S., Kiehlbauch M., Cooperberg D., Aydil E.S. (2005). Feature-scale model of Si etching in SF_6_ plasma and comparison with experiments. J. Vac. Sci. Technol. A Vacuum, Surf. Film.

[88] Institute for Microelectronics, TU Wien ViennaTS, the Vienna Topography Simulator. http://www.iue.tuwien.ac.at/software/viennats/.

[89] Seele S. (2002). Stress and Structure Evolution during Volmer-Weber Growth of Thin Films. Ph.D. Thesis.

[90] Filipovic L., Selberherr S. (2015). Stress considerations in thin films for CMOS-integrated gas sensors. ECS Trans..

[91] Low H.M., Tse M.S., Chiu M.M. Thermal induced stress on the membrane in integrated gas sensor with micro-heater. Proceedings of the 1998 Hong Kong Electron Devices Meeting (Cat. No. 98TH8368).

[92] Coppeta R., Lahlalia A., Kozic D., Hammer R., Riedler J., Toschkoff G., Singulani A., Ali Z., Sagmeister M., Carniello S., van Driel W.D., Pyper O., Schumann C. (2020). Electro-thermal-mechanical modeling of gas sensor hotplates. Sensor Systems Simulations.

[93] Lee S., Dyer D., Gardner J. (2003). Design and optimisation of a high-temperature silicon micro-hotplate for nanoporous palladium pellistors. Microelectron. J..

[94] Spruit R.G., van Omme J.T., Ghatkesar M.K., Garza H.H.P. (2017). A Review on development and optimization of microheaters for high-temperature in situ studies. J. Microelectromech. Syst..

[95] Moridi A., Ruan H., Zhang L., Liu M. (2013). Residual stresses in thin film systems: Effects of lattice mismatch, thermal mismatch and interface dislocations. Int. J. Solids Struct..

[96] Lee D.D., Chung W.Y., Choi M.S., Baek J.M. (1996). Low-power micro gas sensor. Sens. Actuators B Chem..

[97] Phatthanakun R., Deekla P., Pummara W., Sriphung C., Pantong C., Chomnawang N. Fabrication and control of thin-film aluminum microheater and nickel temperature sensor. Proceedings of the 8th Electrical Engineering/Electronics, Computer, Telecommunications and Information Technology (ECTI) Association of Thailand-Conference 2011.

[98] Zhang K., Chou S., Ang S. (2007). Fabrication, modeling and testing of a thin film Au/Ti microheater. Int. J. Therm. Sci..

[99] Xu L., Li T., Gao X., Wang Y. (2010). Development of a reliable micro-hotplate with low power consumption. IEEE Sens. J..

[100] Bhattacharyya P., Basu P., Mondal B., Saha H. (2008). A low power MEMS gas sensor based on nanocrystalline ZnO thin films for sensing methane. Microelectron. Reliab..

[101] Dibbern U. (1990). A substrate for thin-film gas sensors in microelectronic technology. Sens. Actuators B Chem..

[102] Haneef I., Burzo M., Ali S., Komarov P., Udrea F., Raad P. Thermal characterization of SOI CMOS micro hot-plate gas sensors. Proceedings of the 2010 16th International Workshop on Thermal Investigations of ICs and Systems (THERMINIC).

[103] Ali S.Z., Udrea F., Milne W.I., Gardner J.W. (2008). Tungsten-based SOI microhotplates for smart gas sensors. J. Microelectromech. Syst..

[104] Yan W., Li H., Kuang Y., Du L., Guo J. (2008). Nickel membrane temperature sensor in micro-flow measurement. J. Alloys Compd..

[105] Monika D., Arora A. (2013). Design and simulation of MEMS based microhotplate as gas sensor. Int. J. Adv. Res. Comput. Eng. Technol..

[106] Mele L., Santagata F., Iervolino E., Mihailovic M., Rossi T., Tran A., Schellevis H., Creemer J., Sarro P. (2012). A molybdenum MEMS microhotplate for high-temperature operation. Sens. Actuators A Phys..

[107] Balakrishnan V., Dinh T., Phan H.P., Kozeki T., Namazu T., Dao D.V., Nguyen N.T. (2017). Steady-state analytical model of suspended p-type 3C–SiC bridges under consideration of Joule heating. J. Micromech. Microeng..

[108] Creemer J., Briand D., Zandbergen H., Van der Vlist W., De Boer C., de Rooij N.F., Sarro P. (2008). Microhotplates with TiN heaters. Sens. Actuators A Phys..

[109] Benn G.G.S. (2001). Design of a Silicon Carbide Micro-Hotplate Geometry for High Temperature Chemical Sensing. Ph.D. Thesis.

[110] Spannhake J., Schulz O., Helwig A., Krenkow A., Müller G., Doll T. (2006). High-temperature MEMS heater platforms: Long-term performance of metal and semiconductor heater materials. Sensors.

[111] Ali S.Z., De Luca A., Hopper R., Boual S., Gardner J., Udrea F. (2015). A low-power, low-cost infra-red emitter in CMOS technology. IEEE Sens. J..

[112] Lahlalia A., Le Neel O., Shankar R., Selberherr S., Filipovic L. (2019). Improved sensing capability of integrated semiconducting metal oxide gas sensor devices. Sensors.

[113] Siegele M., Gamauf C., Nemecek A., Mutinati G.C., Steinhauer S., Köck A., Kraft J., Siezert J., Schrank F. Optimized integrated micro-hotplates in CMOS technology. Proceedings of the 2013 IEEE 11th International New Circuits and Systems Conference (NEWCAS).

[114] Çengel Y.A., Cimbala J.M., Turner R.H. (2017). Fundamentals of Thermal-Fluid Sciences.

[115] Parameswaran M., Robinson A.M., Blackburn D.L., Gaitan M., Geist J. (1991). Micromachined thermal radiation emitter from a commercial CMOS process. IEEE Electron Device Lett..

[116] März M., Nance P. (2000). Thermal Modeling of Power Electronic Systems.

[117] Murthy K., Bedford R. (1978). Transformation between Foster and Cauer equivalent networks. IEEE Trans. Circuits Syst..

[118] Tulzer G., Baumgartner S., Brunet E., Mutinati G.C., Steinhauer S., Köck A., Barbano P.E., Heitzinger C. (2013). Kinetic parameter estimation and fluctuation analysis of CO at SnO_2_ single nanowires. Nanotechnology.

[119] Fort A., Mugnaini M., Rocchi S., Serrano-Santos M.B., Vignoli V., Spinicci R. (2007). Simplified models for SnO_2_ sensors during chemical and thermal transients in mixtures of inert, oxidizing and reducing gases. Sens. Actuators B Chem..

[120] Degler D., Wicker S., Weimar U., Barsan N. (2015). Identifying the active oxygen species in SnO_2_ based gas sensing materials: An operando IR spectrsocopy study. J. Phys. Chem. C.

[121] Wicker S. (2017). Influence of Humidity on the Gas Sensing Characteristics of SnO_2_: DRIFTS Investigation of Different Base Materials and Dopants. Ph.D. Thesis.

[122] Hua Z., Tian C., Huang D., Yuan W., Zhang C., Tian X., Wang M., Li E. (2018). Power-law response of metal oxide semiconductor gas sensors to oxygen in presence of reducing gases. Sens. Actuators B Chem..

[123] Hua Z., Li Y., Zeng Y., Wu Y. (2018). A theoretical investigation of the power-law response of metal oxide semiconductor gas sensors I: Schottky barrier control. Sens. Actuators B Chem..

[124] Hua Z., Qiu Z., Li Y., Zeng Y., Wu Y., Tian X., Wang M., Li E. (2018). A theoretical investigation of the power-law response of metal oxide semiconductor gas sensors II: Size and shape effects. Sens. Actuators B Chem..

[125] Foo K.Y., Hameed B.H. (2010). Insights into the modeling of adsorption isotherm systems. Chem. Eng. J..

